# Exposure to Language in Video and its Impact on Linguistic Development in Children Aged 3–11: A Scoping Review

**DOI:** 10.5334/joc.385

**Published:** 2024-07-17

**Authors:** Anna Elizabeth Gowenlock, Courtenay Norbury, Jennifer M. Rodd

**Affiliations:** 1 Clinical, Educational, and Health Psychology, University College London, London, UK; 2 Clinical, Educational, and Health Psychology, UCL, London, UK; 3 Experimental Psychology, University College London, London, UK

**Keywords:** Language development, video media, screen time, vocabulary, comprehension

## Abstract

Early exposure to books can benefit language acquisition by expanding children’s linguistic experience and engaging them in a shared activity (Nation et al. 2022; Dowdall et al., 2020). Video media (including television) could potentially fulfil a similar role by exposing children to new linguistic phenomena in an engaging setting. However, while many studies have examined the impact of screen-time on cognitive development (for a review see Kostyrka-Allchorne et al., 2017), the findings relating specifically to language remain unclear. The aim of this review is to understand how encountering language content in video media might impact a variety of language skills in children aged 3–11. This review maps the methods and findings of 93 studies that met preregistered criteria with the goal of understanding which factors impact learning outcomes following video exposure. Results from observational (N = 31) and experimental (N = 62) studies reveal a divided literature in which video viewing is linked to short-term benefits for learning specific linguistic structures from high-quality video media, as well as having negative or null long-term associations with standardised language measures. Results highlight various methodological difficulties and limitations faced by experimental and observational approaches and reveal the importance of video quality and viewing context for language learning.

The primary focus of this review is the relationship between childhood language skills and exposure to video media. This topic is motivated by recent studies reporting a small positive relationship between shared book reading and language skills (Dowdall et al., 2020; Noble et al., 2019). However, despite these positive findings, shared book reading is not an activity that is equally available or beneficial to all families. Access to both books and time may be limited, particularly for the most disadvantaged families. For example, the National Literacy Trust (2023) reported that 40.9% of parents said there was no quiet space for their child to read at home and that they feel too busy (12.4%) or stressed (10.3%) to engage with their child’s literacy at home. As such, it is important to understand how more accessible activities such as television viewing impact children’s language skills. The impact of television and other video media on language skills is a controversial topic. While some researchers present high-quality television as an alternative means to boost language skills (e.g. Rice et al., 1990), others point to evidence linking high levels of screen exposure in childhood to poor language outcomes (Zimmerman & Christakis, 2005). Understanding this relationship is crucially important given the prevalence of viewing among children. Ofcom (2022) report that 81% of UK 4–15 year olds watched live or catch-up TV, and that this age group watched an average of just under six hours of TV (including streaming) per week. If children are able to learn about language from video media, this may represent an opportunity to boost language skills across society. On the other hand, if viewing negatively impacts language skills then this prevalence is concerning. Official guidelines often tend towards the latter view, with the World Health Organization (World Health Organization, 2019) and the American Association of Paediatricians (Council on Communications and Media, 2016) both suggesting that parents limit their children’s screen time, particularly in infancy.

## Negative impacts of video media

There are a number of reasons for this pessimism regarding the relationship between exposure to video media and language skills. Primary among these is the displacement hypothesis (as discussed in Neuman, 1988; and Roberts et al., 1993). This is the idea that time spent watching television displaces other more beneficial activities such as shared book reading or social interaction with a caregiver, therefore reducing other opportunities for children to enhance their language skills. While there is some recent evidence that increased screen-time is associated with decreased sleep (Przybylski, 2019) and social interaction (Putnick et al., 2023) in children, it is not clear whether these relationships are causal, or if they would remain when controlling for factors such as socioeconomic status (see discussion in Hassinger-Das et al., 2020). Furthermore, recent work tends to focus on ‘screen-time’ as a whole, making it difficult to untangle displacement effects of any individual medium.

Another concern comes from findings that very young children do not learn effectively from language content presented over video. Experiments by Kuhl et al. (2003), reported that 9-month-old American infants were able to learn Mandarin phonetic contrasts after interacting with a live speaker but did not learn when the same language content was presented in a pre-recorded video. Similarly, Roseberry et al. (2009) found that a group of 30 to 35-month-old children were able to learn verbs from short video clips when supplemented with live social interaction but not when they were presented with the same video clips alone (by contrast, an older group of 36 to 42-month-olds were able to learn from the video clips alone). While this effect is possibly limited to very young children, it emphasises the importance of social interaction for language development. Indeed, high quality social interaction is thought to be one mechanism by which shared book reading can boost children’s language skills (Mol & Neuman, 2014). Social interaction is inherent in shared book reading whereas television viewing can be a solitary activity, possibly limiting its impact on linguistic development.

## Potential positive effects of video media

However, there are a number of reasons to think that exposure to language in video may be beneficial for children’s language skills. Firstly, children’s television is specifically designed to be engaging and entertaining. Fisch’s Capacity Model (Fisch, 2000) suggests that learning will be enhanced when viewers are interested in the subject matter because this will increase the pool of cognitive resources dedicated to processing the content. Therefore, high levels of engagement and interest in television may provide a useful opportunity to build children’s knowledge and language skills during viewing. According to this theory, learning is also more likely to occur when educational content is embedded centrally within the plot, and when the narrative itself is easy to process, since this allows more cognitive resources to be allocated to processing the educational content (Fisch, 2000). While this model suggests that learning from television is possible, it also suggests that learning outcomes will depend on stylistic and structural features of the programme and that children are likely to learn more from programmes that they enjoy.

Secondly, it has been suggested that engaging media content might act as a social partner for young children, especially when on-screen characters directly address the viewer (Richert et al., 2011). Parasocial relationships (the one-sided relationships that people form with on-screen characters, Horton & Wohl, 1956; Bond & Calvert, 2014) have been the focus of recent studies indicating that children learn best from socially meaningful characters that they trust (Calvert, 2017). Characters in children’s television programmes may directly address the viewer thereby increasing the social contingency between character and viewer (Richards & Calvert, 2017). This may suggest that seeing television as a solitary, non-social activity is misguided, particularly in the case of child-directed programmes, and that children may learn best from programmes that are designed to be interactive.

Finally, watching television and video may widen children’s linguistic environment and expose them to new words and structures. One reason that shared book reading is thought to be beneficial is because the language of books is more complex than child-directed speech in terms of both vocabulary and syntax (Dawson et al., 2021; Montag, 2019; Montag et al., 2015). While the language of television has not been studied as extensively as the language of books, available evidence suggests that subtitles of children’s television shows include key vocabulary items that will be useful to children in the early stages of reading (Green, 2021). It is therefore possible that television could play a similar role to literature in expanding the linguistic environment of young children, both in terms of complexity and diversity of linguistic forms.

## This review

Given the uncertainty surrounding the impact of video media on children’s language skills, the primary aim of this review is to collate and characterise research examining the impact of encountering language through video on children’s linguistic development between the ages of 3 and 11. We chose to use a relatively narrow video media definition, requiring studies to focus on situations in which children watched videos that contained language content. We chose this definition, rather than one that was broader (e.g. ‘screen-time’) or more specific (e.g. television only) because we aimed to include any study that might be informative about what children can learn when they encounter language in video media, regardless of whether this was traditional video media (e.g. television), new media (e.g. YouTube), or experiments in which children watch videos created by the researchers. We chose to limit the review to children under the age of 11 because this is a time in which children’s language skills are still developing rapidly. The lower age limit was chosen because of consistent findings that children below the age of three struggle to process and understand the content of video media and are therefore unlikely to learn language content. For example, Roseberry et al. (2009) found that children over 36 months were able to learn verbs from exposure to video alone but children below 36 months were only able to learn when videos were accompanied by live social interaction. This is supported by a recent review by Jing et al. (2023) which reports that the effect size for the positive relationship between screen exposure and vocabulary was significantly larger for children over 36 months compared with a younger group.

Since the goal here was to map a methodologically diverse and divided literature, we took the approach of conducting a scoping review rather than a systematic review or meta-analysis. Due to the divisive nature of this topic, particular attention is paid to the relationship between methodological choices and findings in order to understand the nature of the evidence that supports opposing viewpoints. A number of previous reviews have examined the effects of screen media on cognitive development in children. However, these reviews either focus on children younger than three (Close, 2004; Linebarger & Vaala, 2010; Richert et al., 2011), use definitions of media exposure that are broader or more specific than that used here (for example, Karani et al. (2022), Madigan et al. (2020), and Richert et al. (2011) include studies with a general measure of ‘screen time’, while Close (2004) and Kostyrka-Allchorne et al. (2017) look only at the impact of television), or include a different set of outcome measures (for example, Kostyrka-Allchorne et al. (2017) look at cognitive development more broadly and Jing et al. (2023) focus entirely on vocabulary). This review is unique in the specificity of the media exposure requirements, the age range of participants, and the diversity of methodological approaches included. This is a crucial first step towards understanding the potential of television and screen media to act as an accessible alternative to shared book reading in boosting children’s language skills.

## Methods

### Protocol

This review was conducted in line with the JBI guidance on scoping reviews (Peters et al., 2020). A protocol for this review was registered on the Open Science Framework (OSF, https://osf.io/) prior to conducting the final search. The protocol can be found at https://osf.io/h43yc/?view_only=438169a9e2ea44648d3fc2a304bc491f.

### Eligibility criteria

Eligibility criteria were defined according to the Participant, Concept, and Context (PCC) approach. A comprehensive list of initial criteria is included in the review protocol and the key requirements for inclusion are outlined below:

#### Participants

Only studies that included children between the ages of 3 and 11 were included in this review. In order to be included, the study needed to include at least 10 children who met the age requirements (i.e. a group of at least 10 children whose mean age was between 3 and 11). In cases where only one group in a study met the age criteria, only the results of that group are included in the review.

The majority of participants in each sample had to be tested in their dominant language. This requirement ensured that studies were focused on L1 development without excluding studies with diverse samples that might include some L2 learners of English (e.g. in a school setting). No restrictions were placed on other participant factors such as developmental diagnoses or SES.

#### Concept

The concepts examined in this review (‘language development’ and ‘video media’) were defined in relation our broader goal of comparing exposure to video media and shared book reading. Firstly, the concept of ‘language development’ examined here is limited to language outcomes which could plausibly be affected by both book reading and video media. This includes receptive and expressive vocabulary, syntactic knowledge, and comprehension, as well as higher level skills such as metaphor and narrative understanding. Studies examining only orthographic knowledge or phonetic/phonological processing are not included in this review. Studies which did not have a numeric outcome measure or that did not report a statistical analysis of their results were excluded. The statistical analysis in included studies had to examine the link between video exposure and language outcomes in some way (e.g. through a comparison of outcomes for different viewing conditions, or by predicting language outcomes from viewing time etc.).

Secondly, the concept of ‘video media exposure’ in this review is restricted to instances where language content is encountered through the medium of video, whether this is through television, film, or video chat etc. Therefore, studies that examine the effects of media without sufficient language content (such as video games or TikTok) are not included in this review, nor are studies that use broad, non-specific measures of ‘screen-time’. These stimuli criteria relate to our research goal of understanding specifically how exposure to language in video media impacts linguistic development. It is this restriction on video media which differentiates the present review from previous work focusing on ‘screen media’ more broadly (Richert et al., 2011; Madigan et al., 2020; Karani et al., 2022).

#### Context

No restrictions were placed on the context in which children were exposed to video. Studies were eligible for inclusion regardless of whether viewing was naturalistic or lab-based. Beyond restrictions on participants, outcome measures, and video media, the criteria for methodological design were kept deliberately broad. This review therefore covers a wide range of potential methodologies from experimental studies that use researcher-created video stimuli, to observational studies that record naturalistic television exposure. This methodological diversity is important to gain a comprehensive picture of the evidence surrounding the impact of video media on language development.

#### Types of evidence

This review considered only primary studies that have been published in journals or as doctoral theses. Grey literature was not included.

#### Amendments to protocol

Eligibility criteria were amended with these clarifications following discussions between the researchers conducting the title and abstract screen:

- Records that are not in English should be excluded.
- Screen time measures can be collected before the age of three so long as the language outcome measure is collected while the children are between 3 and 11 years old.
- Papers in which vocabulary measures are used as a proxy for other skills (such as socio-emotional skill or science knowledge) can be included.
- Studies in which learning necessarily relies on reading skill (e.g. studies in which D/deaf children watch videos with subtitles) should be excluded.

### Search strategy

Six online databases were searched: PsycInfo, LLBA, ERIC, Scopus, Web of Science, and Medline. The same search terms were used across all databases and included terms related to language learning (e.g. ‘language development’, ‘language acquisition’, ‘vocabulary’, ‘word learning’, ‘syntax’), terms related to screen media (e.g. ‘video media’, ‘television’, ‘screen use’), and terms relating to children and childhood (e.g. ‘child*’, ‘primary school’, ‘kindergarten’). Search terms were combined with logical operators such that papers had to contain references to language AND screens AND children. Searches were conducted on titles, abstracts, and keywords but not on the full-text. No restrictions were placed on date of publication. The full list of search terms and operators is given in Appendix I, along with the search history for each database.

The search was conducted in February 2023. Across the six databases the search returned 1997 results. Results were downloaded as .RIS files and imported into Rayyan (Ouzzani et al., 2016) for deduplication and screening. Rayyan detected 1067 possible duplicates, of which 703 were confirmed as duplicates and removed leaving 1294 records for abstract screening.

### Selection process

#### Title and abstract screening

The first stage of the selection process was the title and abstract screen. During this stage, the titles and abstracts of all non-duplicate records were compared against the eligibility criteria and either excluded from the review or sent to full-text screening. Any titles and abstracts which lacked sufficient information to make a final decision were sent to full-text screening.

R Studio (R Core Team, 2022) was used to select a random subset of 10% of the papers (130 papers) to be screened independently by two researchers. Agreement between researchers on this subset was 90.77%. Any conflicting decisions were discussed and resolved. Most disagreements related to the screen time criteria and whether there was sufficient information in the abstract to reject papers based on their measures of screen time. Since overall agreement was above the 90% agreement threshold specified in the protocol, the remaining records (1164) were screened by a single researcher. The majority of records that were removed at this stage were either the wrong publication type (e.g. book chapter, conference proceedings), or did not meet criteria for study population or language outcome measure. 145 records were identified for full text screening at this stage.

#### Full-text screening

**Figure 1:**
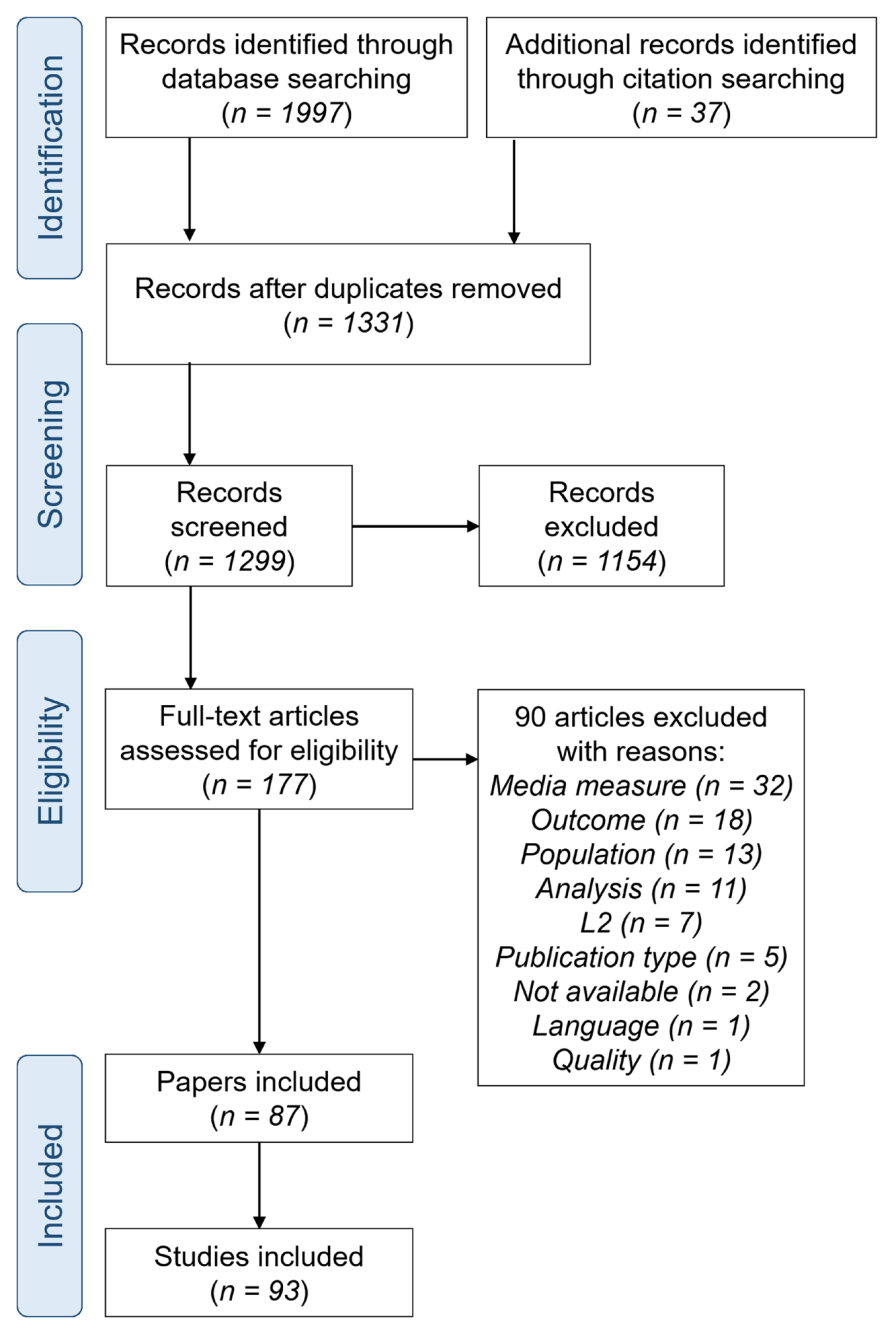
PRISMA flow diagram.

During full-text screening records were checked against the eligibility criteria and final decisions were made about their inclusion. R studio (R Core Team, 2022) was again used to select a random 10% of records (15 records) for screening by a second researcher. Agreement was 80.00% (below the 90% threshold specified in the protocol) so any disagreements were discussed and a further 10% of records were screened by both researchers. Agreement for this second subset was 93.33% and so the remaining records were screened by a single researcher following discussion of any disagreements. Disagreements related to outcome measures (e.g. whether they relate to language or general knowledge), analysis procedure (whether the link between viewing and outcomes was examined), and media exposure (whether the measure related to video specifically or screen time more broadly). A total of 66 records were identified for inclusion at this stage. See the PRISMA-flow diagram for further information (Figure 1).

#### Additional studies

Following the screening of records returned in the original database search, the reference lists of included studies were manually checked for any other relevant sources. A list of possible additional sources was created on the basis of titles and then abstracts and full-texts were checked against the eligibility criteria. An additional 32 records were identified at this stage and 23 studies from 21 papers were included in the final set.

### Data extraction

Extensive methodological detail was extracted from all included studies so that we could create a comprehensive picture of the ways that researchers have studied the impact of video on language. Broadly this involved recording basic publication meta-data, sample characteristics, video stimuli information, outcome measures, aims, hypotheses, and findings. Studies were also classified as ‘experimental’ or ‘observational’: experimental studies included all studies in which researchers manipulated an independent variable, including quasi-experimental methods in which it is impossible to randomly assign participants to conditions (as in studies comparing clinical and typically developing children) and studies in which exposure to video media is controlled by the researcher but no comparisons are made between conditions. Observational studies included designs in which the independent variable is not manipulated by researchers including descriptive survey studies and observational association studies. Different methodological information was recorded for each study classification. For experimental studies, more detail was collected about control conditions, stimuli, and whether viewing was interactive. For observational studies, more detail was collected about different age groups, screen-time measures, and whether children watched with a co-viewer or alone. The planned data extraction tool can be found in the protocol.

Categories were added or refined in the early stages of data extraction if it became clear that further detail was necessary to answer the research question. For this reason, further categories were added relating to the timeframe of the study, sample SES, the scope of outcome measures, the type of experimental manipulations, and the quality of observed media exposure.

### Synthesis

Data analysis (including descriptive statistics and visualization) was done in R (R Core Team, 2022. The code used to calculate descriptives and generate graphs can be found at: https://osf.io/h43yc/?view_only=438169a9e2ea44648d3fc2a304bc491f. While basic descriptive information was examined for all types of study, in-depth synthesis was conducted separately for experimental and observational studies due to the numerous differences in method and focus between these two categories. For experimental studies, synthesis included a qualitative analysis of the type of stimuli that were used and a quantitative analysis of the frequency of different experimental manipulations. For observational studies, the focus was on the types of screen time measures that were used and the degree to which studies measured quality or type of viewing in addition to quantity. For both study types, an analysis of outcome measures was carried out. The number of studies that used a particular category of measure was calculated and differences between the two study types were explored. In addition, each observational study was coded according to whether they reported a significant increase in children’s language skills in response to video exposure, a significant decrease, or mixed or null findings. Experimental manipulations were coded according to whether they had a significant effect on the different outcome measures used. While this is not a meta-analysis and is not intended as a conclusive assessment of the evidence, this analysis allows us to understand the general picture of findings that are presented in this field.

## Results

### Studies identified

A total of 87 records were identified for inclusion in this review. 94.6% of included records were published journal articles and 5.4% were doctoral theses. Within these 87 records there were 93 individual studies that met the inclusion criteria. Extracted data from these 93 studies is available in the supplementary materials on OSF (https://osf.io/h43yc/?view_only=438169a9e2ea44648d3fc2a304bc491f). This file contains basic information for each study (including time and place of publication, information about participants, etc.) as well as detailed information relating to study design such as outcome measures and findings for each study, experimental factors and stimuli (experimental studies only), and details of screen time measures (observational studies only).

### Overview of studies

#### Year of publication

**Figure 2:**
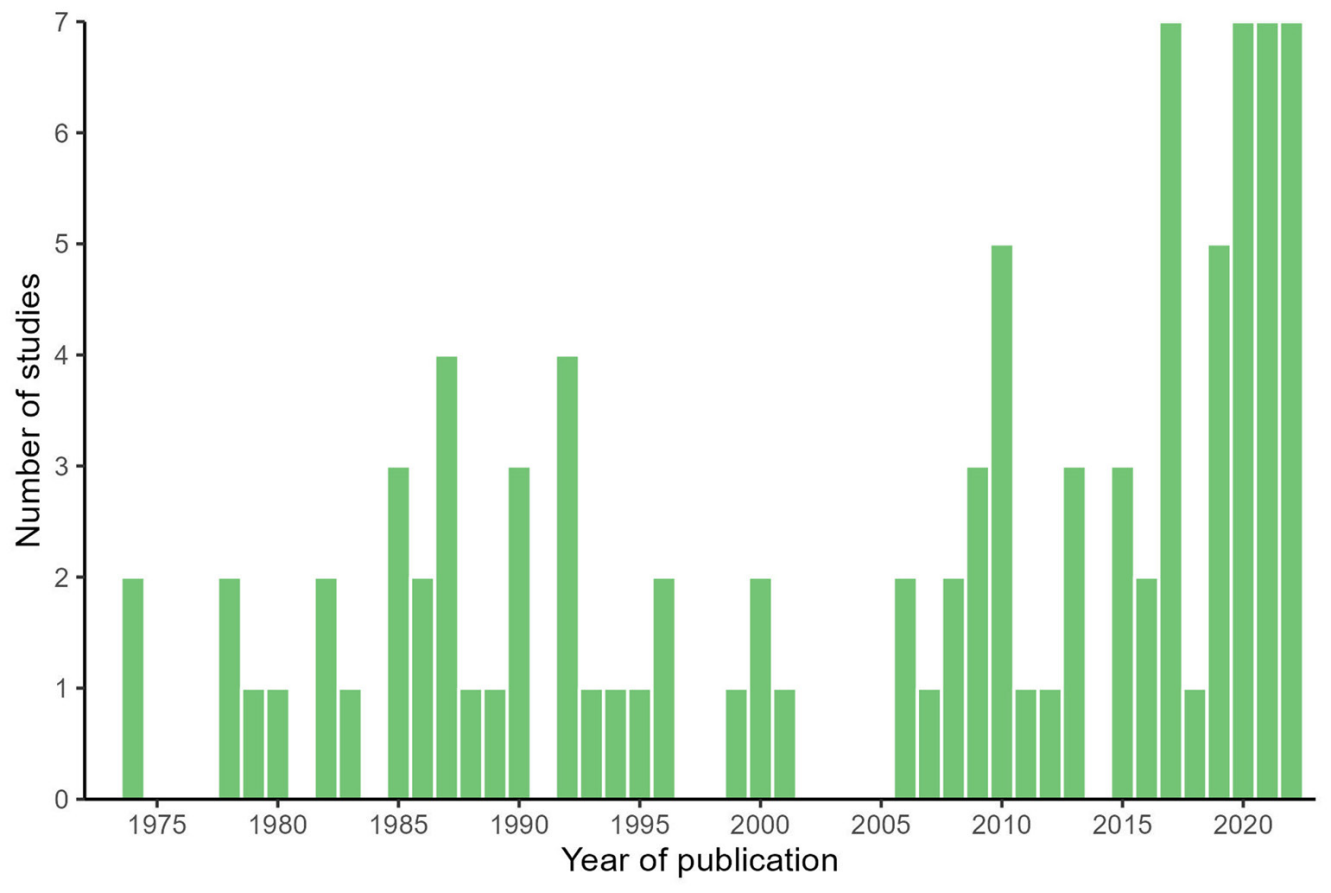
Publication year for all 87 papers included in the review.

Most studies (61.3%) were published within the last 20 years. The earliest studies included in this review were conducted in the 1970s and there was a cluster of related studies published in the late 1980s and early 1990s, mainly focusing on the ability of children with ADHD to process language in TV clips. Figure 2 shows the distribution of studies over time.

#### Countries

The majority of studies (74.2%) were conducted in the USA. The remaining studies were conducted in Canada, China, Iran, Australia, Israel, France, Korea, the Netherlands, Taiwan, Turkey, Scotland and the UK.

#### Study design

The studies covered a broad range of methodologies including word learning experiments, intervention studies, longitudinal association studies, corpus analyses, and case-control designs, with the most frequent designs being learning experiments and longitudinal association studies. Sixty-two studies (66.7%) were classed as experimental (meaning the stimuli and viewing setting were controlled and children were usually randomised to different conditions which were later compared). Thirty-one (33.3%) were classed as observational (meaning that video viewing was not manipulated or controlled but rather occurred in a naturalistic setting and was measured in some way by the researcher). While in most cases this classification was straightforward, two difficult cases emerged. Both Perez (1990) and Neulight (2012) instructed participants to watch specific video content and tested participants on stimuli specific language outcomes. However, neither study made any comparison between viewing conditions or included any control group. For the purposes of this review these studies have been classified as experimental although they do not sit neatly in either category. Results from the two study design categories are presented separately below.

#### Study timeframe

Seventy-six (81.7%) studies collected data in a single wave or time point. Seventeen (18.3%) of studies collected data over at least two longitudinal waves. Within longitudinal studies, one key point of variability was the relative timing of the video exposure measures and the testing of language outcomes. Some observational studies related early TV exposure to later language development (e.g. Schmidt et al., 2009) while others interleaved multiple measurements of video exposure and language development over a number of years (e.g. Rice et al., 1990). Across all 93 studies, 26 (28.0%) included at least two cohorts at different ages at any one time point, while 67 (72.0%) included only one cohort.

### Participants

Sample size for individual studies ranged from 12 to 5682. The majority of studies had samples smaller than 200 (71.0%) and only five studies (4.7%) tested fewer than 25 participants. The average sample size was much larger for observational studies (Mean = 750.10, Median = 173.0) than for experimental studies (Mean = 120.73, Median = 78.5).

#### Age

**Figure 3:**
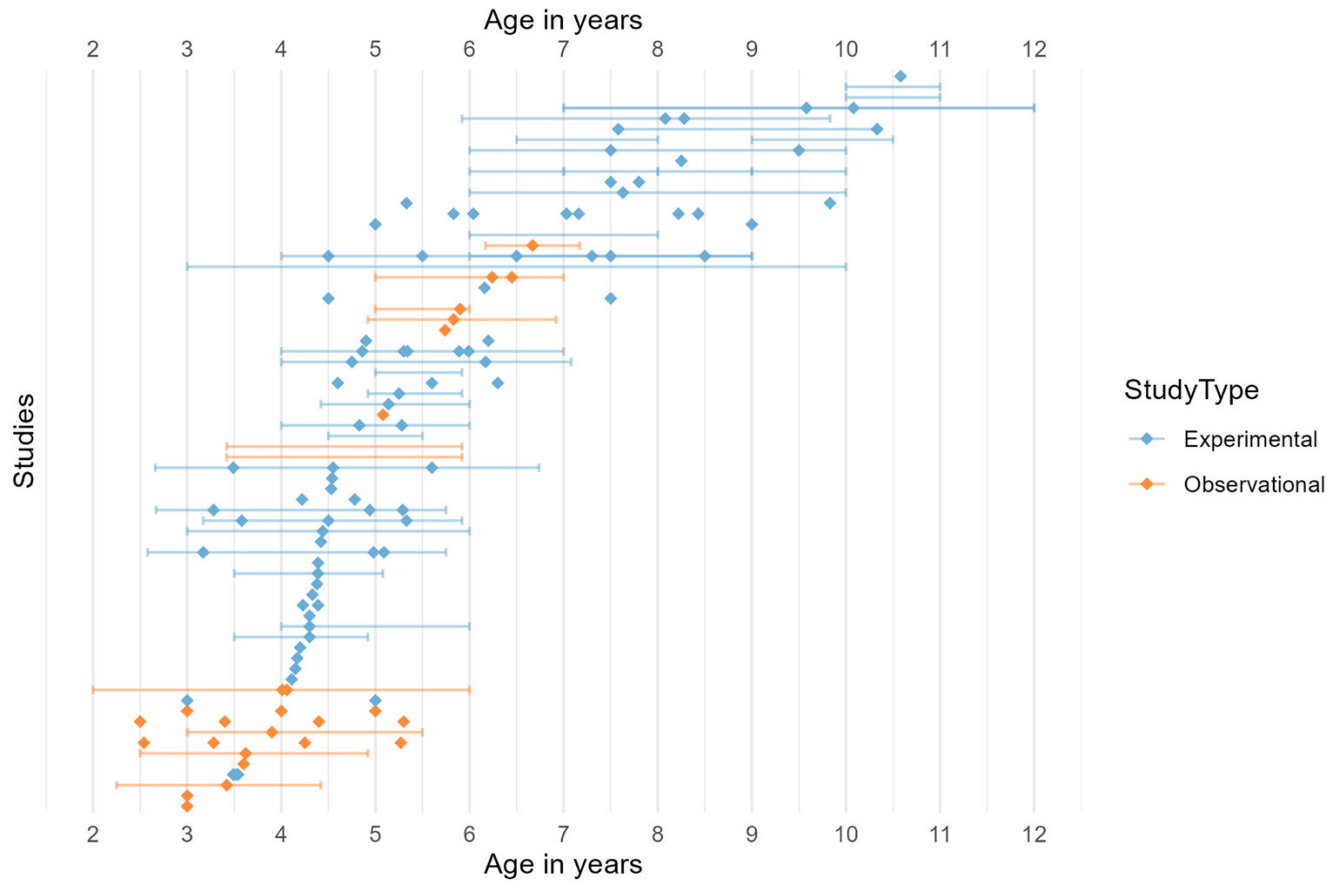
Age data (means and ranges) from single-timepoint studies. Each diamond refers to the mean age of a group of participants in a particular study. Lines show the age range of each group (where reported). Colour refers to study design: blue for experimental studies and orange for observational studies. Note that where a study has reported age data for two groups separately, the means for each group are plotted separately on the same row.

**Figure 4:**
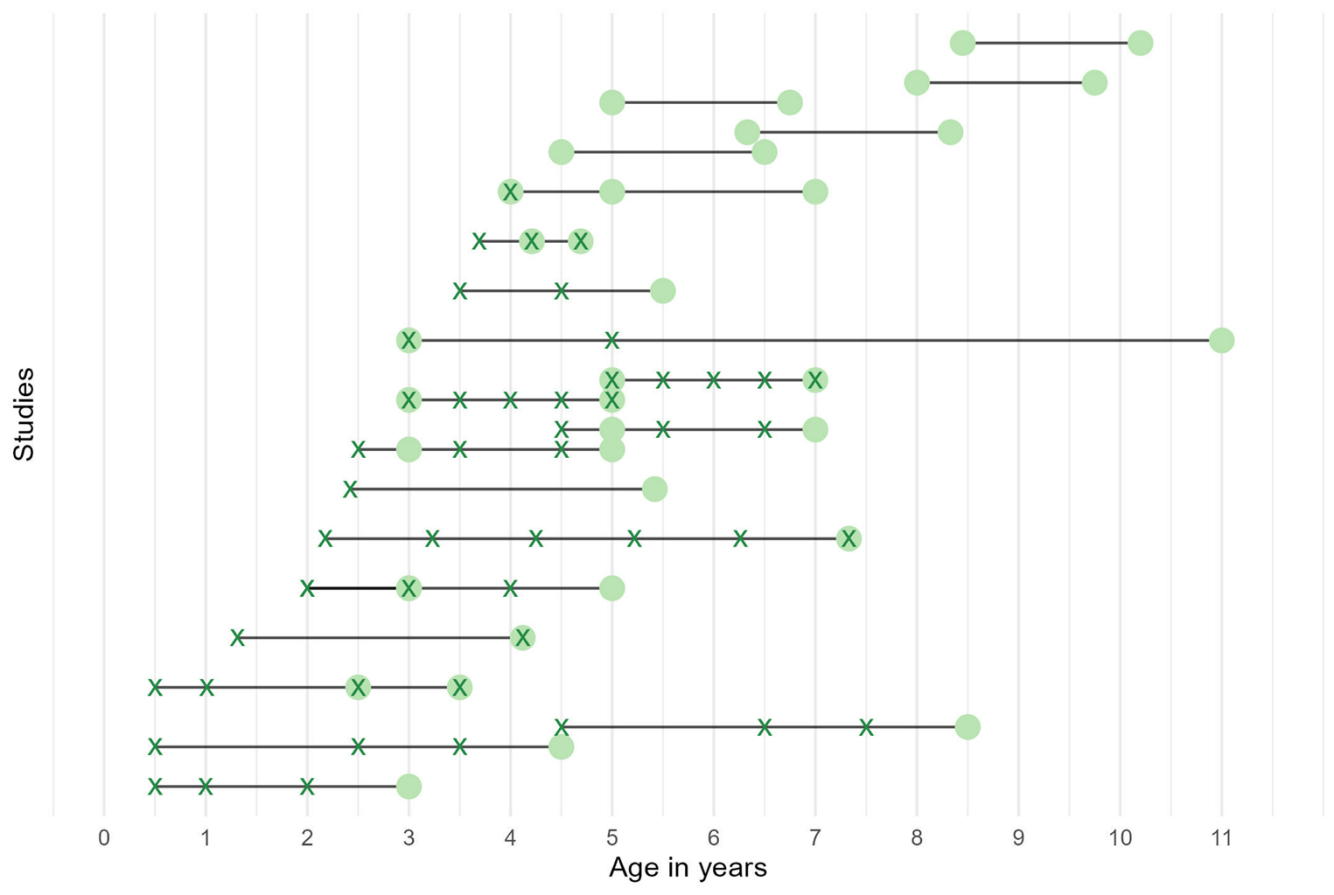
Participant ages during different waves of longitudinal studies. In this graph, each row represents a single longitudinal study and black lines represent the ages covered by the study from start to finish. Where a study contains two cohorts, these are represented separately as offset lines. Each green cross represents the age at which screen time data were collected, while each green dot represents the age at which language data were collected.

The inclusion criteria required that at least one group of participants in each study be between 3 and 11 years of age. Studies were found across the entirety of this range. The majority of studies focused on children in preschool and early primary school (around 3–6 years). There was inconsistency in the reporting of participant ages, with some studies reporting only a range and others reporting only a mean. Figure 3 shows available age data for single time point studies. Included within single time point studies are short intervention studies in which measures are repeated within a matter of months, but in which the main goal of the study was to assess response to an intervention or condition rather than to assess development or age effects. Note that in cases where studies included multiple cohorts, only those groups with a mean age between 3–11 are included in the graph. Figure 4 shows age data for longitudinal studies split according to whether researchers were collecting data about TV exposure or language skills at each time point. In the case of longitudinal research, studies met the age criterion so long as language measures were taken between 3–11, regardless of the timings of video exposure assessments. Therefore, eight longitudinal studies in this review included screen time measures taken between the ages of 6-months and 2.5 years and one study included a language measure taken between 2 and 3 years (Stockdale et al., 2022). Only two studies looked at the association between viewing measures taken exclusively before the age of three with language outcomes at three or above (Schmidt et al., 2009; Pagani et al., 2013).

#### Socio-economic status (SES)

Twenty-two studies (20 of which were experimental) recruited participants from low SES backgrounds and three deliberately sampled children from both high and low SES backgrounds. SES information was not reported in 24 studies (all experimental).

#### Developmental Diagnoses

The majority of included studies focused on typically developing children. Five studies (5.4%) reported that their sample included a minority of participants with developmental conditions (although this was not necessarily a variable of interest), while 12 studies (12.9%) deliberately recruited participants with developmental conditions in order to address research questions related to developmental language disorder, autism, or ADHD. Almost all of these studies recruited typically developing children as a comparison group (although in Kim et al. (2008) 100% of participants had been diagnosed with Down Syndrome).

#### Language

The majority of studies (81.7%) assess native English speakers. Other languages included Mandarin, Persian, French, Hebrew, Korean, and Turkish. Two studies assessed balanced bilingual children in two languages (English and Spanish, Van Horn & Kan, 2016; Dutch and Frisian, Bosma & Blom, 2020). Although the inclusion criteria required studies to assess the majority of participants in their native language, studies varied in terms of the percentage of native speakers and the percentage of multilinguals.

### Outcome measures

The inclusion criteria allowed for a wide range of outcome measures related to spoken language skill. For the purposes of this review these assessments have been categorised according to the linguistic level that they are measuring. Categories were defined for four linguistic levels (concepts, vocabulary, syntax and morphology, and narrative) with an additional category for composite or holistic measures which targeted multiple linguistic levels (*General*). Measures were also categorized as *Targeted* or *Generalised* according to their scope. *Targeted* measures are those that assess knowledge of specific language forms that had appeared within the video stimuli, while *Generalised* measures used generic test items that hadn’t necessarily appeared during video exposure. As such, *Targeted* measures were generally used to assess learning of specific forms, while *Generalised* measures were generally used to assess linguistic development more broadly. Definitions for each of these outcome measure categories, as well as example measures for each category, are given in Table 1 below.

**Table 1:** Categories of outcome measures.


LANGUAGE LEVEL	CATEGORY DEFINITION	EXAMPLE TASKS GENERALISED	TARGETED

Conceptual knowledge	Tests of underlying conceptual knowledge (not necessarily linked to a particular linguistic form)	Tests of conceptual content of children’s language (unrelated to recently encountered video content). *Example: Measuring the amount of semantic information present within an utterance [42]*.	Tests of concepts recently encountered in video. *Example: Asking children to point to a picture of a concept underlying a word without linking it to the wordform (e.g. ‘point at the picture that ‘tells musicians what to do’ for the concept of ‘conductor’) [12]*.

Vocabulary	Tests of knowledge of, or familiarity with, individual word forms or meanings.	Tests to assess overall vocabulary level. *Example: Standardised vocabulary tests such as the Peabody Picture Vocabulary Test (PPVT) [32]*.	Tests of words recently encountered in video. *Example: Asking children to point at pictures representing the meanings of words they have just encountered [2]*.

Syntax or morphology	Tests of syntactic or morphological knowledge that focus on linguistic structure rather than meaning.	*Example: Counting the number of different syntactic forms produced [42]* *Example: Test of Dutch plural formation [34]*.	*N/A*

Narrative	Measures related to children’s understanding or recall of longer linguistic units such as narratives. Includes assessments of basic narrative recall as well as higher level understanding (i.e. inferred or implied information).	Tests of knowledge of story structure or inferencing ability (unrelated to recently encountered video content) *Example: Asking children to arrange a set of pictures and then tell a story based on the pictures [10]*.	Tests of recall or inferencing based on a recently encountered video *Example: Free recall of story events in a short video clip [68]*. *Example: Arranging pictures to the plot of a video clip [36]*.

General	Tests that span a number of linguistic levels to provide a composite score that reflects a child’s overall level of ‘language ability’.	*Example: Standardised tests of language level such as the Test of Early Language Development (TELD) [20]*.	*N/A*


*Note*: Numbers in square brackets indicate the ID number of a paper that uses the example measure. These can be matched to papers by finding the PaperID column in the extracted data on OSF. Cells are marked as N/A where there were no outcome measures in that category.

The majority of studies also reported additional outcomes which were either unrelated to language (e.g. information about sleep patterns or physical activity) or that were related to language but did not meet the criteria for inclusion (e.g. outcomes relating to orthographic knowledge). Only oral language outcomes are presented here.

**Figure 5:**
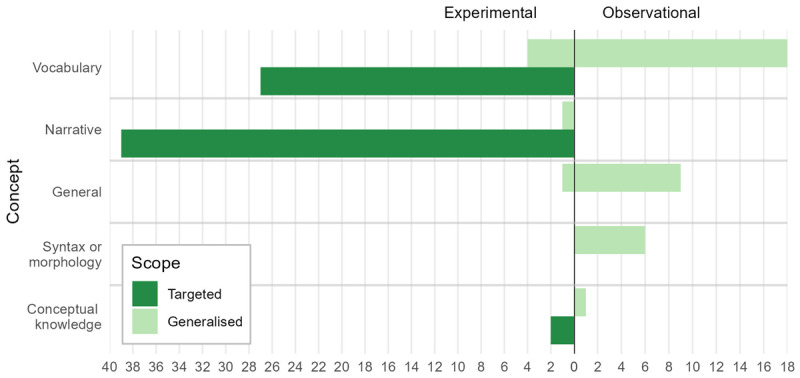
Outcome measures for all studies. This figure shows the number of studies that measured a particular language skill (i.e. Vocabulary) of a particular type (targeted vs generalised). Outcomes used by experimental studies are on the left, observational studies are on the right. Note that each study can contribute only once to each count, so an observational study that reported two generalised vocabulary measures (for example) would only be counted once in this category.

Figure 5 displays the number of experimental and observational studies that use a particular measure (e.g. *Targeted Vocabulary*). The clearest imbalance between the two study designs relates to the scope of the measures. No observational studies used targeted measures (although this would be difficult, it would be possible, e.g. by testing knowledge of linguistic forms that were known to have appeared during naturalistic viewing) and very few experimental studies used generalised measures. This reflects differences in the aims of the studies: experimental studies were mainly designed to assess specific learning processes while observational studies were mainly designed to identify associations between viewing behaviours and general linguistic development. The second clear difference between the two study types is that narrative measures were frequently employed in experimental studies, but were not used by any observational studies. Most observational studies did not place restrictions on the types of programmes that children were watching so it would have been difficult to test children’s understanding of the specific narratives they encountered (e.g. by using targeted narrative measures). However, it is not clear why none of the included observational studies examined the effects of viewing on generalised narrative skills.

Detailed findings relating to the different outcome measures are reported separately for experimental and observational studies in the following sections.

### Experimental studies

#### Experimental stimuli

The type of video stimuli used in experimental studies varied greatly from episodes of real children’s TV shows (e.g. ‘The Rugrats’ in Lorch et al., 2010; ‘Sesame Street’ in Hayes & Kelly, 1985; and ‘Dora the Explorer’ in Calvert et al., 2007), to videos created by researchers for the purpose of their study (e.g. Nussenbaum & Amso, 2016; Schryer et al., 2015; and Gaudreau, 2021). Some researchers used a hybrid approach and edited real children’s videos to meet the requirements of the study, for example by replacing the original audio with new narration containing certain target words (e.g. Rice & Woodsmall, 1988), or by removing participatory cues from original stimuli (e.g. Calvert et al., 2007). Regardless of the stimuli source, video clips were often explicitly described as being educational in nature (49.2% of experimental studies) and in all but one case the stimuli were designed for a child audience (Collins et al. (1978) used an episode of a programme designed for a general audience). Individual episodes/clips were typically quite short and ranged from 21.42 seconds (Neuman et al., 2019) to 45 minutes (Mares, 2006, Study 1). Usually individual clips were only viewed once by each child but sometimes they were repeated up to four times (Mares, 2006, Study 2).

The amount of information about the language content of the stimuli varied greatly across studies. Word learning studies that embedded target words in video narratives typically provided information about the target words such as word class or frequency (e.g. Rice & Woodsmall, 1988; Neuman et al., 2021). These words included nouns, verbs, adjectives, and adverbs and were often selected to be low frequency to increase the likelihood that they were unknown to participants (e.g. ‘dragonfly’ and ‘enthusiastic’ in Neuman et al., 2021). Sometimes information was provided about the linguistic context surrounding target words. For example, Neuman et al. (2019) classified the ostensive linguistic cues that introduced target words into categories such as ‘definitions’ (e.g. “A subway is an underground train”, p.35) or ‘features of target words’ (e.g. “Otters have webbed feet that help them swim”, p.35). Other studies manipulated broader language features across conditions, for example by embedding questions directly addressing the audience (e.g. Nussenbaum & Amso, 2016) or including additional explanatory segments (e.g. Calvert et al., 1987). Studies that manipulated the overall narrative structure or style typically included some additional information describing each condition (e.g. ‘traditional’ narratives in Linebarger & Piotrowski (2009) were defined as following “a simple linear story with a problem and a solution that unfolds over the episode”, p.53, while ‘difficult’ narratives in Campbell et al. (1987) were those that “contained more nonimmediate narration. more abstract words … more use of passive voice, and less repetition”, p.316). In general however, researchers typically did not provide information about the language content of their stimuli beyond those language features that were deliberately manipulated (e.g. vocabulary items, narrative style) or general information about the style of the programme (e.g. ‘child-directed’ or ‘educational’). Notable exceptions to this include Linebarger & Piotrowski (2010) who analysed narrative structures and learning strategies in six 30-minute programmes, and Neuman et al. (2019) who analysed vocabulary scenes and pedagogical supports in 200 episodes of programmes that were designed to improve children’s language and literacy skills. Linebarger & Piotrowski (2010) found that expository programmes contained more complex language and fewer strategies to improve comprehension than narrative programmes, but they do not provide information about the specific language used. Neuman et al. (2019) report that two thirds of the episodes they analysed included vocabulary instructional importunities and that the vast majority (96%) of targeted vocabulary scenes focused on teaching nouns (Neuman et al., 2019).

#### Experimental designs and independent variables

Broadly, the results of the experimental studies in this review can be organised according to three fundamental research questions:

1. Do children learn language from video?
2. Do children learn better from video compared with other media?
3. What factors modulate children’s language learning from video?

The following section presents the findings of included experimental studies organised by these three questions. In each case, we give an overview of the types of study designs that have been used as well as a summary of the results.

##### Do children learn language from video?

Some studies were specifically designed to investigate target word-learning following exposure to video stimuli. A number of studies compared pre- and post-test target vocabulary scores and report that children had significantly higher scores after exposure to video with the target words embedded (Perez, 1990; Rice et al., 1992; Rice et al., 1994; Neulight, 2012; Strouse et al., 2013; Gev et al., 2017; Hseuh et al., 2017; Neuman, Samudra, et al., 2020; Neuman et al., 2021). Significant differences in post-test target vocabulary scores were also reported between groups who viewed videos containing the target vocabulary words and groups who did not – either because they viewed an edited version of the video that did not contain the target words, or because they did not view any video stimuli (Rice & Woodsmall, 1988; Rice et al., 1992; Rice et al., 1994; Oetting et al., 1995; Schryer et al., 2015; Gev et al., 2017; Hsueh et al., 2017; Gaudreau, 2021). A number of studies also assessed programme specific target word-learning by comparing scores on vocabulary outcomes to chance level. Performance above chance on at least one post-viewing vocabulary measure was reported by Neuman, Flynn, et al. (2020); Samudra, Wong, et al. (2019); Samudra, Fynn, et al. (2019); and Nussenbaum and Amso, (2016). These findings were sometimes limited to particular contexts or measures. For example, the youngest children tested by Nussenbaum and Amso (2016) did not perform above chance in low-interactivity conditions, and Neuman, Flynn, et al. (2020) did not find above chance performance on a measure of understanding words in a new context. Taken together, the studies above suggest that children can learn target vocabulary words that are embedded in video stimuli.

Three studies tested for improvements in generalised vocabulary measures following video exposure. Schryer et al. (2015) report significantly improved scores on a generalised vocabulary measure for a group of children who were exposed to an animated book reading intervention but not for children who did not view the videos. In the remaining two studies, there were significant improvements from pre- to post-test in vocabulary scores but there were no group effects (Farah et al., 2019; Strouse et al., 2013). This makes it difficult to untangle the effects of the intervention from improvements due to time or repeated testing. More specifically, Farah et al., (2019) found significant improvements over time for both a group that received a shared book reading intervention and a control group who viewed videos of the same stories, while Strouse et al. (2013) report significant improvements over time for four groups of children who all viewed the same video stories with varying levels of additional scaffolding. These studies therefore do not provide clear evidence of improved general vocabulary scores following exposure to video.

Findings about the impact of video on narrative skills were less consistent. Linebarger & Piotrowski (2009) tested the effects of exposure to 40 episodes of television programmes in three different narrative styles on generalised measures of narrative skill and story knowledge. Narrative skills were assessed by asking children to retell a story from a wordless picture book and answer comprehension questions about it. Narrative production was assessed by asking children to arrange pictures in order and then tell a story using the pictures. These measures are therefore intended as indices of children’s knowledge of stories and narratives generally, rather than measures of their comprehension of specific video content. There is some evidence of improved narrative skills following viewing, with significant improvements from middle to post-test on explicit and implicit comprehension and story knowledge measures for children viewing traditional narratives. However, these results are complicated by the finding that the non-viewing group also improved significantly over time on a number of measures. Better performance was found on all measures for children who viewed either traditional or embedded narratives rather than expository narratives or no viewing, but no individual condition resulted in consistently better performance on all measures. It is therefore unclear from these results whether exposure to stories in video can improve children’s general narrative skills. The remaining studies that measured narrative outcomes are not designed to test for improvements in narrative production or understanding following exposure to video. Instead, these studies tested children’s recall or understanding of specific stories presented through video.

##### Do children learn better from video or other media types?

While the majority of experimental studies examined children’s responses to video media only (43, 69.4%), the remaining 19 studies (30.6%) compared outcomes following exposure to video and another media type such as books or radio. Most commonly, the same stories were presented in each format (e.g. by creating a storybook based on the video) to control for story effects (e.g. Neuman et al., 2021; Meringoff, 1980; and Terrell & Daniloff, 1996). In some cases, media type was manipulated as a within-subjects variable, usually by asking children to watch one story in video format and then listen to a second story in another media type (e.g. Neuman et al., 2017). In other cases, a between-subjects design was used in which some children watched a story in video format while others heard a story in a different medium (e.g. Meringoff, 1980). Both cases allow for an assessment of whether learning differs when stories are presented through video or other mediums.

Beginning with the studies that examined the effect of media type on targeted vocabulary, there were three studies that reported a main effect of media type. Firstly, Terrell and Daniloff (1996) asked children to either watch a short story in the form of an animated cartoon, listen to the same story while viewing a series of still images from the video on a computer screen, or listen to the same story being read to them by the researcher (again accompanied by the same still images from the cartoon). They found that children performed significantly better in the book-reading condition compared to the other two media conditions on targeted vocabulary measures including auditory-verbal recognition (identifying the target word that they heard in the story from a list of three words) and auditory-visual recognition (word-to-picture matching). No differences were reported between the video and still image conditions. The two other studies reporting a main effect of media type were both published in Neuman et al. (2021). While the first and second studies published in this paper find no significant differences in target word learning between a video condition and a live storybook reading condition, the second and third studies additionally report a media effect wherein groups who were exposed to the same medium twice (e.g. watching two videos or reading two books) had significantly lower scores on a receptive vocabulary measure compared to groups who were exposed to a story in each medium (e.g. a video followed by a book).

In contrast, six studies that compared vocabulary learning across different media types found no significant main effects or interactions involving different media conditions. These studies compared either pre-existing television clips (Goldstein et al., 2022; Neuman et al., 2021), short animated stories (Van Horn & Kan, 2016; Neuman et al., 2017), or videos of researchers reading books aloud (Gaudreau, 2021; Farah et al., 2019) to a range of alternate media types including live storybook reading (Neuman et al., 2021; Van Horn & Kan, 2016; Neuman et al., 2017; Gaudreau, 2021; Farah et al., 2019) or interactive play sessions (Goldstein et al., 2022). Most commonly researchers used targeted tests of vocabulary that had appeared in the stimuli, although Farah et al. (2019) instead report scores on a standardised test of vocabulary (the WPPSI). No significant differences between media conditions were reported for any of these tests. The media effects reported in this review therefore do not provide clear evidence that vocabulary learning is more or less successful in one medium over another, with only one study showing significantly worse performance in the video condition.

Media type was also manipulated by 13 studies that assessed narrative recall. These studies tended to compare the amount of story information that children were able to recall after exposure to stories in different media. Six of these studies found significant main effects of media type on narrative measures. In three cases these effects indicated a video advantage with children making fewer recall errors after watching a video vs listening to a radio clip (Hayes et al., 1986), and recalling more information when stories were presented as videos rather than audio clips (Gibbons et al., 1986) or storybooks read by the researcher (Beentjes & van der Voort, 1993). Two studies reported mixed results, with benefits for video for recall of actions (Meringoff, 1980) and story order and details (Beagles-Roos & Gat, 1983) but benefits for recall of figurative or expressive language from books (Meringoff, 1980) and radio (Beagles-Roos & Gat, 1983). Finally, Kendeou et al. (2008) report a significant effect of media type (books vs TV) on narrative recall, but do not specify the direction of the effect.

Seven studies reported no significant main effects or interactions involving media type for narrative recall outcomes. These studies compared results following exposure to video clips (either clips from TV shows or films (Neuman et al., 2021; Kim et al., 2008; Hayes & Kelly, 1985; Neuman, 1989; Neuman, 1992), animated storybooks (Neuman et al., 2017), or a video of the researcher reading a storybook (Gaudreau, 2021)) vs exposure to other media (either books read aloud by the researcher (Neuman et al., 2021; Neuman et al., 2017; Gaudreau, 2021; Neuman, 1989), audio clips of stories (Kim et al., 2008; Hayes & Kelly, 1985) or books read by the children themselves (Neuman, 1992)). None of these studies found significant condition differences for measures of recall of story content (Neuman et al., 2021, Neuman et al., 2017, Kim et al., 2008; Gaudreau, 2021; Hayes & Kelly, 1985; Neuman, 1989), story sequencing (Neuman et al., 2017) or inferencing (Gaudreau, 2021; Neuman, 1992). The findings relating media type to narrative measures are therefore more variable than those for vocabulary measures, with some evidence that children perform better on particular aspects of narrative recall depending on the media type. It is important here to bear in mind that it is very difficult to separate recall of linguistic and visual content in measures of narrative recall as children may be remembering visually presented story information. Therefore, even when comparing video to picture books, any observed differences in recall could be influenced by the differing amounts of visual information in each medium and aren’t necessarily due to better or worse processing of linguistic information. So, while some studies report ‘better recall’ of content presented via video, this doesn’t allow for strong conclusions about *linguistic* comprehension specifically.

##### What factors modulate children’s language learning from video?

**Figure 6:**
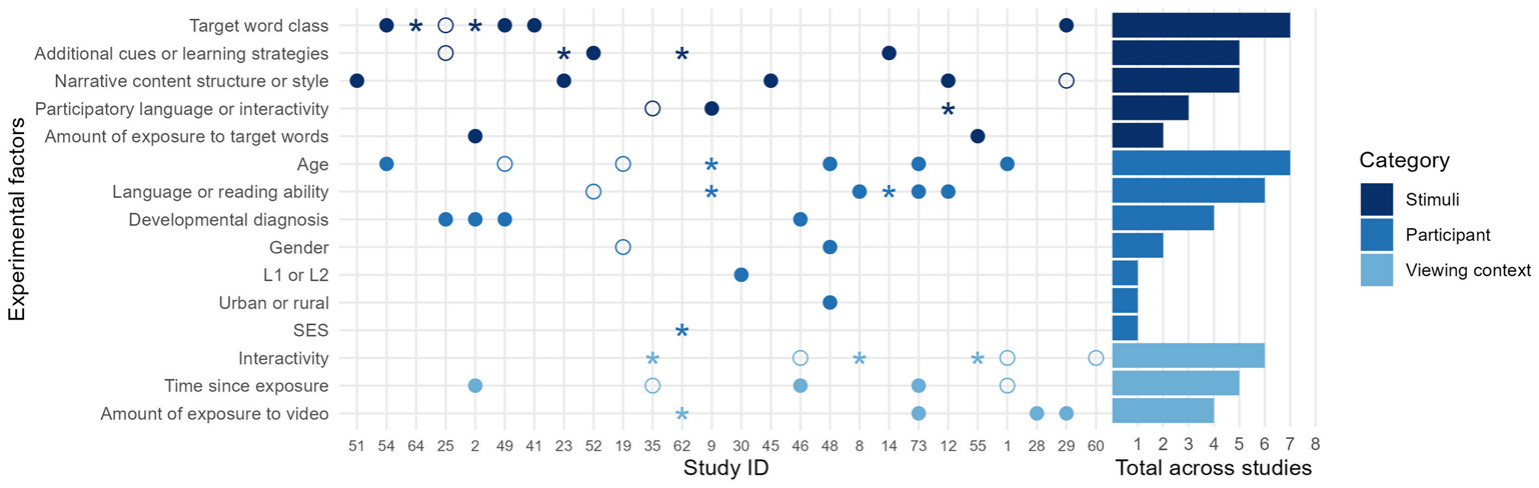
Effects of experimental factors on vocabulary outcomes. Each point on the x-axis represents a separate study, organized by year of publication (see the StudyID column in the extracted data on OSF to match these numbers to particular studies) and each point on the y-axis indicates a different type of experimental factor. Factors are split into three categories (factors relating to the participant, stimuli, or viewing context). The bar chart on the right indicates the total number of experimental studies that examined a particular factor. Filled circles represent a significant main effect of that factor on at least one vocabulary outcome within a study, asterisks represent a significant interaction or simple effect on at least one vocabulary outcome in the absence of a significant main effect, and empty circles represent no significant effects on any vocabulary outcomes within a study.

**Figure 7:**
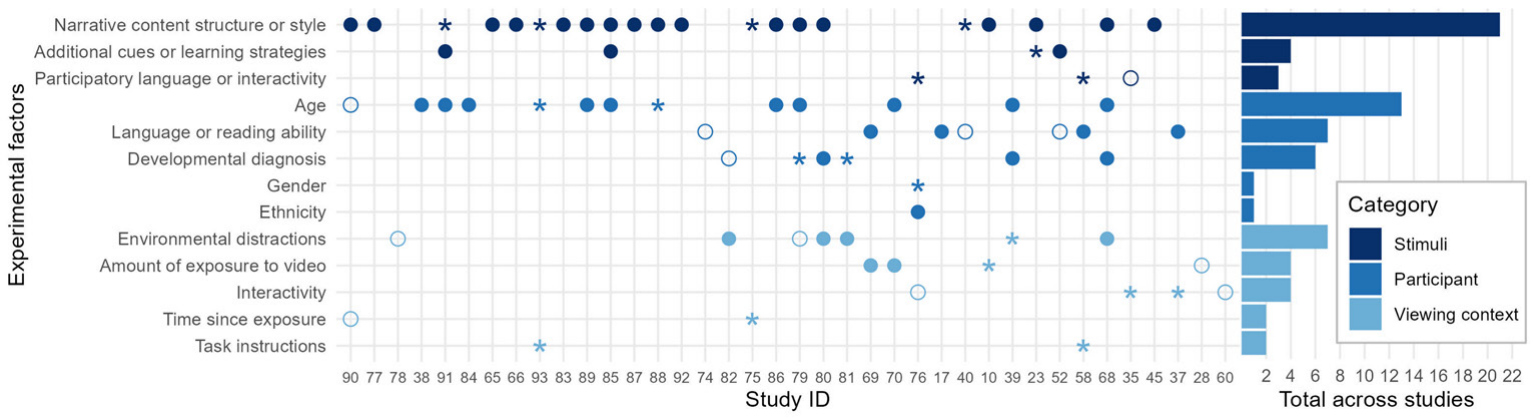
Effects of experimental factors on narrative outcomes. Each point on the x-axis represents a separate study, organized by year of publication (see the StudyID column in the extracted data on OSF to match these numbers to particular studies) and each point on the y-axis indicates a different type of experimental factor. Factors are split into three categories (factors relating to the participant, stimuli, or viewing context). The bar chart on the right indicates the total number of experimental studies that examined a particular factor. Filled circles represent a significant main effect of that factor on at least one narrative outcome within a study, asterisks represent a significant interaction or simple effect on at least one narrative outcome in the absence of a significant main effect, and empty circles represent no significant effects on any narrative outcomes within a study.

Almost all experimental studies examine the factors that moderate learning by comparing outcomes between different participant groups, between manipulations of stimuli features, or between viewing environments. Figures 6 and 7 give a summary of the experimental factors included in different designs (along the y-axis) in different studies (along the x-axis), and whether these factors had a statistically significant effect on outcome measures (significant main effects on at least one outcome measure are indicated by a filled circle, interactions by an asterisk, empty circles indicate no reported effect). For example, Rice et al. (1994) find a main effect of amount of exposure to target words (with better performance for the group who had more exposures) but no main effect of target word class. Since there were a number of interactions involving target word class, this is marked on the graph with an asterisk. Figure 6 shows results for the studies that measured vocabulary while Figure 7 shows results for studies that measured narrative outcomes. Note that for each study, we only include those factors whose effect on the outcome was tested, and that an individual study may appear in both figures if both vocabulary and narrative measures were examined. Factors that were included as a covariate, but were not part of the research questions or hypotheses of a given study are not included in the figures. Additionally, secondary outcome measures which were used to predict language outcomes (e.g. visual attention while viewing) are not included in this analysis.

##### Studies with vocabulary outcomes

Beginning with the studies that measured vocabulary outcomes (Figure 6), we can see that it was most common for researchers to examine factors related to the video stimuli. Target word class (e.g. noun, verb, adjective, etc.) was manipulated by seven studies by embedding words of different classes within the scripts of short animated video clips (as in a paradigm developed by Rice & Woodsmall, 1988). While target word class does appear to impact learning in individual studies, the direction of reported effects in the literature is not consistent. For example, while two studies report an advantage for verbs (Perez, 1990; Rice et al., 1994), three studies report that performance on verbs was significantly lower than at least one other word class (Oetting et al. 1995; Terrell & Daniloff, 1996; Neuman et al., 2021). One study reports no effects of word class (Rice et al., 1992).

The effects of additional cues (e.g. text captions or pauses) or learning strategies (e.g. referencing prior knowledge) were investigated by five studies, again with mixed results. Captions were associated with better performance on a target vocabulary comprehension post-test (Linebarger et al., 2013) in one study, but another reports better scores in a low-SES group when text was absent (Linebarger et al., 2010). Adding a pause before target words had no effect on vocabulary learning (Rice et al., 1992). Other strategies such as adding attention-directing cues led to improved scores but not on all vocabulary outcomes measured (Neuman et al., 2019). It is not clear from this pattern of results that any specific cues or learning strategies are linked to consistently better outcomes.

Narrative content, structure, or style was manipulated by five studies in total. Again, no clear pattern of results emerges from these manipulations. One study embedded target vocabulary items in at least two different stories to check whether the learning of target words was dependent on them being embedded in a specific story or if learning would occur regardless of story context (Neuman et al., 2021; no effect of story). Another tested different target words embedded in four different stories that were presented either through video or live storybook reading to try and understand whether differences in learning related more to the story content or the medium of presentation (Neuman et al., 2017). There was a main effect of story suggesting that story content affected children’s ability to learn target words (although note that in this case, story was confounded with target word features as different words were embedded in each story). Both Neuman, Flynn, et al. (2020) and Linebarger and Piotrowski (2010) manipulated narrative style by comparing expositories (programmes that ‘used multiple vignettes and programme hosts to educate, inform, or describe a particular topic”, Linebarger & Piotrowski, 2010, p.1585) to narratives (programmes that “used a set of story events … to deliver prosocial or academic content”, Linebarger & Piotrowski, 2010, p.1585) and report opposite patterns of results.

Finally, three studies examined the impact of embedding participatory language or interactivity within the video stimuli themselves. This typically involved on-screen characters that would attempt to directly engage the audience by asking questions and by leaving pauses for responses. Of the three studies that looked at this factor, two report simple effects indicating better performance on targeted vocabulary measures when levels of interactivity were higher (Neuman, Flynn, et al., 2020; Nussenbaum & Amso, 2016). Finally, Strouse et al. (2013) compared a viewing condition in which an on-screen actress engaged in dialogic questioning techniques throughout the story to a series of viewing conditions where the clip was played without the on-screen actress (further condition differences are discussed in the ‘viewing environment’ section below). This dialogic actress condition was compared to a condition in which the video itself did not contain participatory language but parents were encouraged to use dialogic questioning techniques while their children were viewing. There was no significant difference between these conditions suggesting that children had a similar response to an interactive adult on video and live parent interaction. The paucity of evidence relating to stimuli interactivity again limits the conclusions that can be drawn.

Finally, two studies examined the effect of amount of exposure to target words. In both cases this involved comparing groups who saw versions of the same video with target words embedded a different number of times (Rice et al., 1994; Neuman, Samudra, et al., 2020); both of these studies report a main effect of repetition wherein more exposures lead to better learning.

The links between participant factors and vocabulary learning were examined by 16 studies in total, with more consistent results than were observed for stimuli factors. The most common participant variable was age, with four studies reporting that older children performed significantly better on at least one targeted vocabulary measure (Rice & Woodsmall, 1988; Hsueh et al., 2017; Samudra, Wong, et al., 2019; Goldstein et al., 2022; Nussenbaum & Amso, 2016). Neulight (2012) and Oetting et al. (1995) find no effect of age after controlling for pre-test scores suggesting that baseline knowledge of target vocabulary may be more important for learning than age. Similarly, five studies report better word learning abilities for children with higher initial vocabulary levels as tested with standardised measures (significant main effects: Samudra, Flynn et al., 2019; Samudra, Wong, et al., 2019; Neuman, Flynn, et al., 2020; significant simple effects within conditions: Nussenbaum & Amso, 2016; Neuman et al., 2019). Linebarger et al. (2010) was the only study to report no significant effect of reading ability on their target word comprehension measure.

Of the studies that included developmental diagnosis as a factor, all report better vocabulary learning in a typically developing control group than the group with a developmental diagnosis. Three studies compared children with and without a diagnosis of developmental language disorder (Rice et al., 1992; Rice et al., 1994; Oetting et al., 1995). In the fourth study, children with autism were shown to have lower initial performance on a test of emotion vocabulary than peers, but after viewing a programme that contained target emotion vocabulary, both groups performed similarly (Gev et al., 2017). Effects of age, language ability, and developmental diagnosis are therefore fairly consistent and in the expected direction.

The remaining participant factors (gender, L1 vs L2, geographical area, and SES) were only examined in a few studies. Two studies examined the impact of gender on targeted vocabulary measures with inconsistent results (Neulight, 2012; Hsueh et al., 2017). One study examined language background and found larger gain scores when children were tested in their second language (Van Horn & Kan, 2016). One study reported an effect of geographical area wherein children from an urban area improved more from pre- to post-test on a targeted measure of vocabulary than children from a rural area (Hsueh et al., 2017 – although note that geographical area may be confounded with SES here). One study reported an SES interaction whereby SES moderated the effect of on-screen print on vocabulary with the on-screen print leading to significantly worse performance for children in the lower SES group only (Linebarger et al., 2013).

The final category of experimental factors relates to the context in which children were exposed to video stimuli. For vocabulary outcome measures, this commonly involved a manipulation of the interactivity of the viewing environment (e.g. co-viewing with a researcher or parent) leading to inconsistent results. Three studies found no effect of interactivity on targeted vocabulary measures (Goldstein et al., 2022; Gaudreau, 2021; Gev et al., 2017) and three report significant interactions involving interactivity. In an examination of the effect of co-viewing, Samudra, Flynn, et al. (2019) found that only children with lower initial PPVT scores benefited from co-viewing an educational programme with the researcher whereas children with higher PPVT scores performed equally well with or without a co-viewer. Similarly, Neuman, Samudra, et al. (2020) found that co-viewing with a researcher only benefitted learning of expressive vocabulary in a low repetition condition in which children heard each target word only 3–4 times, but had no impact on learning when target words were repeated 8–11 times. Finally, Strouse et al. (2013) report that children performed better on a test of target vocabulary in a dialogic questioning condition where parents were instructed to use dialogic questioning techniques (e.g. prompting their children to talk about what was happening in the story), compared to a condition in which parents were instructed to direct their child’s attention to the screen without asking questions, or a condition in which parents were not instructed to interact with their child at all during viewing. These findings suggest that co-viewing and interactivity of the viewing context are not universally beneficial for target vocabulary learning.

Time since exposure was manipulated by six studies. In all cases this involved a comparison of an immediate post-test to a delayed retention test. In three studies, there was a significant main effect of testing time, with scores improving from immediate to delayed test on measures of target emotion vocabulary (Gev et al., 2017, three month delay) and video-specific vocabulary (Rice et al., 1994, one to three day delay, significant improvement only seen for age-matched typically developing control children; Samudra, Wong, et al., 2019, one week delay, significant improvement for expressive targeted vocabulary measure only). It is possible that these effects are due to a simple effect of time or repeat testing since the same items were used across all time points so children would be encountering the test words for the third time in the delayed test. In the final two studies, there was no main effect or interaction involving test time, indicating that scores did not differ significantly from post-test to delayed test (Strouse et al., 2013, two week delay; Goldstein et al., 2022, two week delay). No studies reported a significant drop in performance at delayed test, suggesting that children did not forget target words. This may be due to the relatively short amount of time between immediate and delayed test or intensive exposure to stimuli (10 minutes per day over eight weeks in the case of Gev et al., 2017).

Finally, four studies manipulated the amount of video that children are exposed to by comparing groups who watched different numbers of repetitions of video stimuli. Although the details of this manipulation differed across studies, there was a fairly consistent finding that children performed better when they had more exposure to video stimuli (e.g. compared to a group who had fewer repetitions as in Samudra, Wong, et al., 2019).

##### Studies with narrative outcomes

Figure 7 shows the experimental factors in studies that measured narrative outcomes. As with studies of vocabulary, the majority of these studies manipulated something about the video stimuli. Twenty-one studies manipulated something about the narrative content (e.g. testing recall for different stories), structure (e.g. comparing recall for events that differed in number of causal connections or hierarchical structure within the narrative), or style (e.g. comparing recall of entertainment programmes vs educational programmes). In all cases, narrative manipulations resulted in a significant main effect or interaction. While the details of the effects vary between studies, a number of generalisations can be made. Firstly, there is a consistent finding that children are better at recalling information that is central to the plot compared to peripheral information (Friedlander et al., 1974; Calvert et al., 1982; Lorch et al., 1987; van den Broek et al., 1996; Lorch et al., 1999; Kim et al., 2008; Lorch et al., 2010). Secondly, children appear to be sensitive to the style of the narrative, with better recall for videos that were child-directed and entertaining rather than educational (Campbell et al., 1987; Lorch et al., 2000). Differences in recall were also reported when comparing narratives vs expositories and when comparing different types of narratives such as traditional or embedded narratives (Linebarger & Piotrowski, 2009; Linebarger & Piotrowski, 2010), however, the direction of these effects was not consistent and varied for different types of outcome measure. Effects of narrative content, structure, or style were frequently moderated by participant factors such as age or developmental diagnosis, with older and typically developing children being more sensitive to narrative manipulations (Calvert et al., 1982; Lorch et al., 1987; Lorch et al., 2010).

A number of additional cues or learning strategies were found to have a positive impact on specific narrative outcomes. For example, Linebarger et al. (2010) find a positive effect of on-screen print for literal but not inferential comprehension, while Calvert et al. (1982) found that the salience of visual cues positively impacted recall of central, but not incidental, story content. Findings relating to participatory language or interactivity within clips were inconsistent, with one study finding no difference between a co-viewing condition and a participatory language condition (Strouse et al., 2013), while two others found improvements linked to participatory language only for subgroups of their samples (Calvert et al., 2007; Piotrowski, 2010). The small number of studies that explore the effects cues, learning strategies, and participatory language on comprehension, as well as inconsistencies between these studies, make it hard to draw strong conclusions about the benefits of these stimuli characteristics.

The effects of participant variables on narrative outcomes generally mirrored results for vocabulary. All significant effects of age indicate that older children perform better than younger children (although note that Friedlander et al. (1974) report no difference between four- and five-year-olds). Where there were significant effects of language or reading ability, these indicated that children with higher initial vocabulary or story knowledge performed better overall on measures of recall (Kendeou et al., 2008; Samudra et al., 2020; Piotrowski, 2010) and inferencing (Mares, 2006). Although, note that three studies reported no effects of vocabulary level (Kendeou et al., 2008; Neuman, 1992) or reading risk status (Linebarger et al., 2010) on inferencing skills. All studies examining effects of developmental diagnosis compared children with ADHD to peers without ADHD. While four studies report overall better recall performance in the comparison group, two find that there are group differences only in certain conditions (e.g. when distracting toys are present, Lorch et al., 2000), and one reports equal performance between groups on a measure of free recall (Landau et al., 1992). Only one study examined gender and ethnicity as part of the main research questions (Calvert et al., 2007). They report that Caucasian children did better than Hispanic children on central content recall, but that Hispanic girls benefited more from viewing interactive media than other children.

A common manipulation of context involved comparing narrative scores when viewing a video in the presence or absence of distracting toys. Significant main effects indicate better performance by children who viewed in the absence of toys (e.g. Lorch et al., 2000), while significant interactions generally indicate that the presence of toys increased differences in performance between children with and without ADHD (e.g. Bailey et al., 2009). The effects of amount of exposure to video were inconsistent: positive effects of video repetition were found for story comprehension (Mares, 2006, study 2) and close inferences (Mares, 2006, study 1) but had no reported effect for far inferences (Mares, 2006, study 1) and Linebarger and Piotrowski (2009) report improved performance with more exposure on their implicit comprehension measure only and only for children viewing narratives that followed a traditional narrative structure.

Similar to the results for vocabulary, the effects of interactivity of the viewing context were inconsistent for narrative outcomes. Strouse et al. (2013) report simple effects showing that children whose parents engaged in dialogic questioning while viewing had higher comprehension scores, while Gaudreau (2021) finds no difference in comprehension between a live storybook reading group and a group who watched a pre-recorded video, and both Calvert et al. (2007) and Samudra et al. (2020) report significant effects of co-viewing only for children certain characteristics such as high verbal ability or high attention.

Few studies explored the effects of time since exposure (Beentjes & van der Voort, 1993; Friedlander et al., 1974) or task instructions (Piotrowski, 2010; Field & Anderson, 1985) and none reported significant main effects on narrative measures, making it difficult to draw conclusions about the impact of these variables.

### Observational studies

#### Video exposure measures

Video exposure measures were categorised according to the data collection method, the source of the data, whether they included information about co-viewing (whether the child watched alone or with others), the target audience of viewed content (child-directed or adult-directed), information about the style or content of viewed shows (i.e. genre or language content), and whether children were actively watching or not (foreground vs background TV). While studies may have included these extra contextual factors in their video exposure measure, it was not always the case that this information was used in the statistical analysis (e.g. Farangi and Mehrpour (2022b) collected data about the main programs watched but then did not include that information in their model).

**Figure 8:**
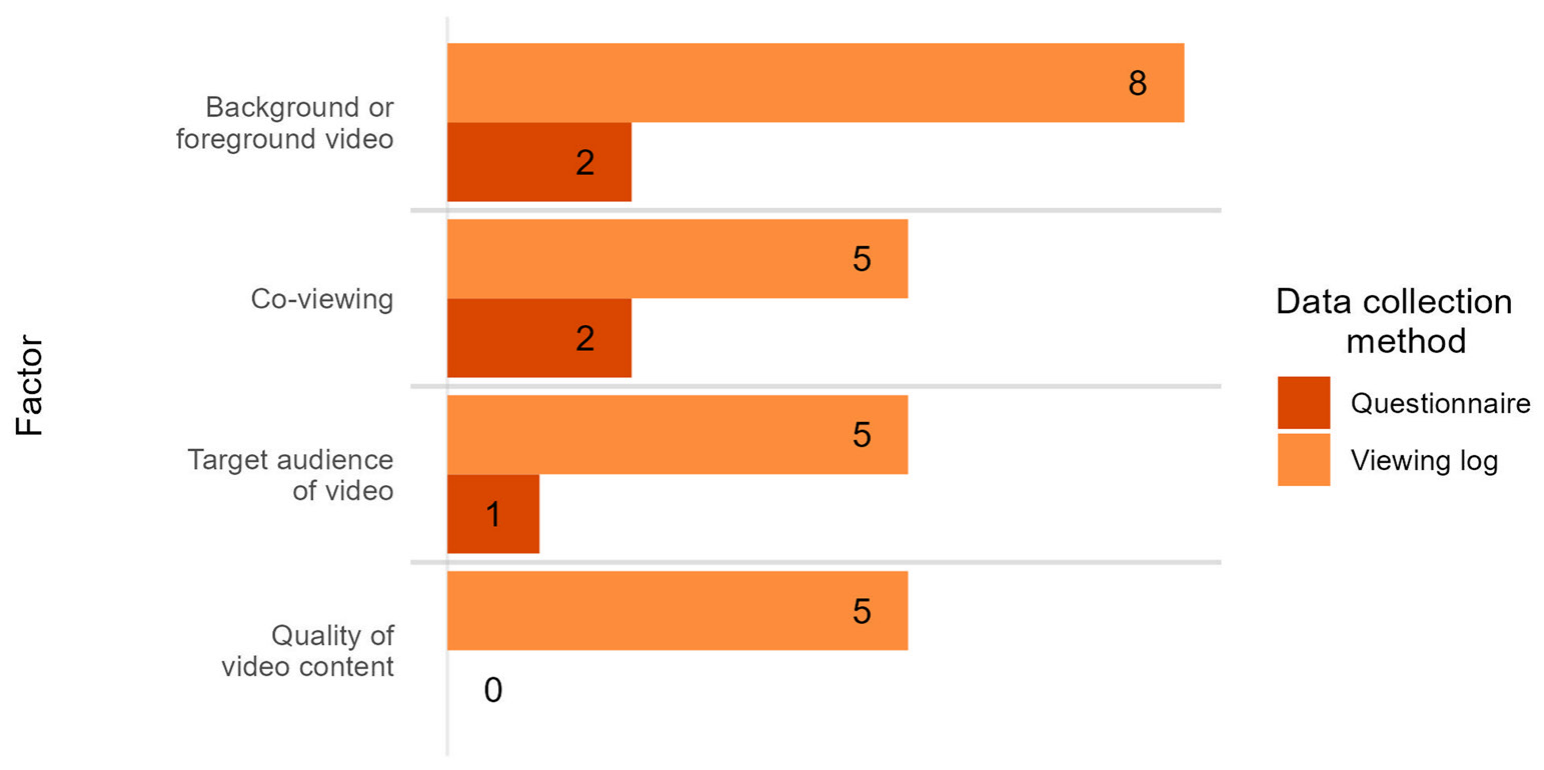
Additional information collected along with viewing data (observational studies only). The y-axis represents the categories of additional information that was measured by different studies, split by type of measurement tool used (questionnaire or viewing log). The x-axis indicates the number of separate studies that measured each factor.

Twenty-one studies (67.7%) used a questionnaire to gather information about video exposure, nine (29.0%) used a viewing log or diary, and one (3.2%) did not report how the data were collected. None used direct observation to measure video exposure. Data was almost always provided by parents although in one case children filled in their own viewing logs (Allen et al., 1992) and in another, the source of the data is unclear (Oryadi-Zanjani et al., 2021). Questionnaires often consisted of one or two simple questions asking parents to recall their children’s typical video exposure or their exposure over a certain timeframe (e.g. over the past week or month). The format of viewing logs was variable but usually parents were asked to record or recall how much time their child spent watching TV over a day or a week. Sometimes parents were asked to record extra details such as the type of programme that was being viewed (Arraf, 1990) or who was present while the child was watching (Barr et al., 2010). Figure 8 displays the number of studies that recorded certain additional details about the viewing context as well as quantity of viewing. Additional contextual information was collected much more often when data were collected via a viewing log rather than a questionnaire.

The majority of observational studies provide no additional information about the specific content that children are watching, including the linguistic features of that content. However, the five studies that measured ‘quality’ in some way do provide some additional information about the content of the programmes. Three of these studies make only broad distinctions between different types of programmes (e.g. commercial/noninformative vs educational/informative TV in Arraf (1990) and Wright et al. (2001); child vs adult-directed in Rice et al. (1990) and Wright et al. (2001)). Only two studies directly assessed the linguistic content of the programmes that children were watching (Selnow, 1978; and Selnow & Bettinghaus, 1982). In both cases the authors used the Developmental Sentence Scoring measure to assess the grammatical complexity of different types of programmes that were popular at the time. In both papers, they report that the most complex language occurred in action dramas, followed by situation comedies, educational shows, family dramas, and cartoons (Selnow, 1978; and Selnow & Bettinghaus, 1982). Beyond these studies, the linguistic content that children are exposed to while viewing is rarely discussed in any great detail.

#### Outcome measures and findings

**Figure 9:**
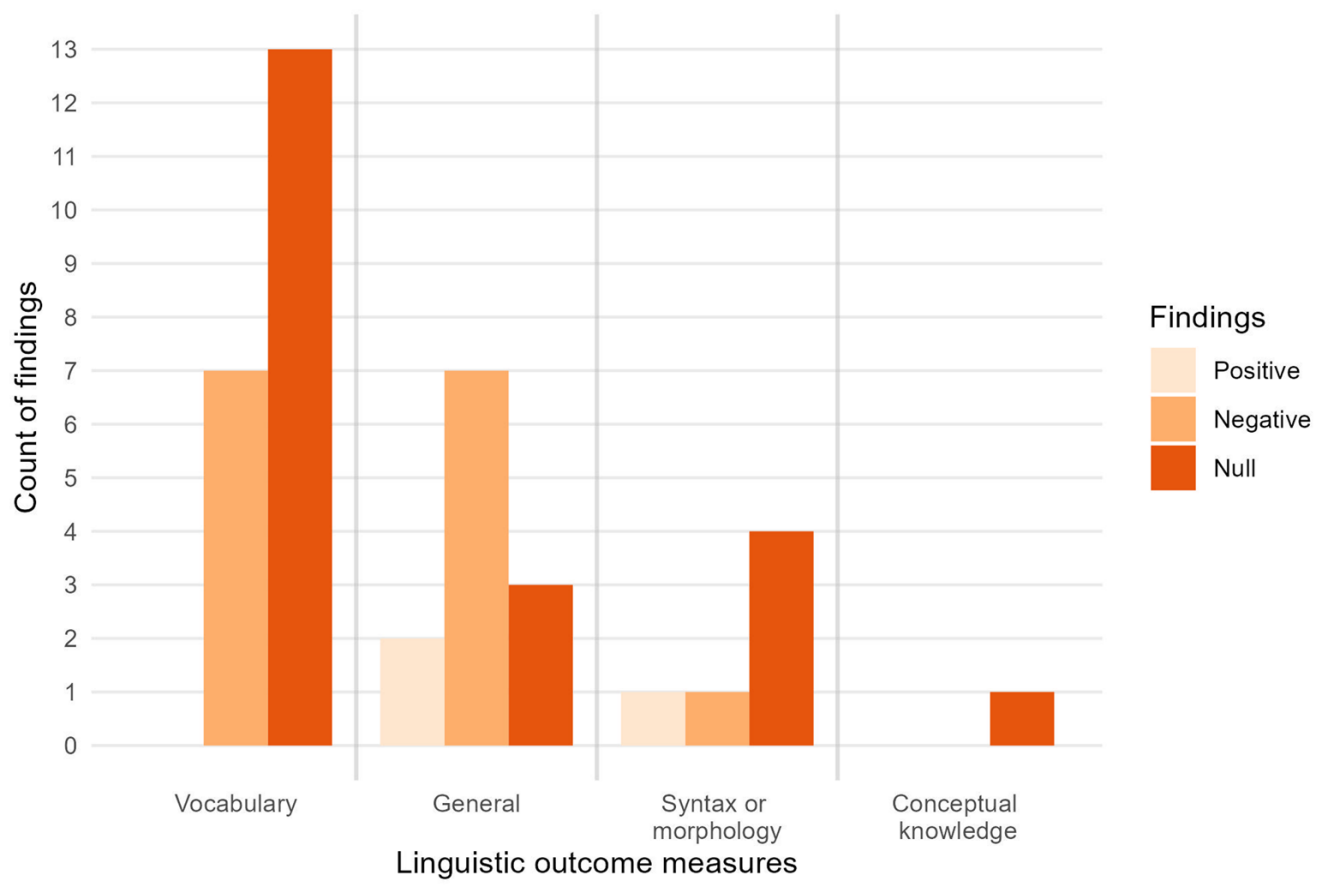
Associations between measures of total video exposure and linguistic outcomes (observational studies only). Different linguistic concepts are represented along the x-axis and the different colours represent different types of association.

Findings from observational studies are presented according to type of outcome measure. These studies all used generalised measures of language, rather than testing knowledge of specific linguistic content that had appeared in video stimuli. Figure 9 shows reported associations between video exposure and outcomes across studies. Note that this graph contains only those studies that took a measure of ‘total video screen time’ and excludes studies that examined effects of more detailed measures such as amount of background TV exposure, or amount of parental co-viewing. This graph displays reported associations between individual screen time measures and individual outcome measures. Therefore, a single study may contribute multiple data points if they report more than one *viewing time – language outcome* pairing or if the same measures are taken at multiple time points. Results of both total video screen time measures and more focused video measures are described below.

##### Vocabulary

As with experimental studies, vocabulary was the most commonly assessed outcome measure in observational studies (18 studies, 58.1%). These studies used a wide range of standardised vocabulary measures (e.g. the *Peabody Picture Vocabulary Test* (PPVT)), or vocabulary subtests from broader measures (e.g. the *Wechsler Preschool and Primary Scale of Intelligence* (WPPSI)). There were no positive associations reported between measures of overall video screen time and generalised vocabulary outcomes. Studies which categorised participants according to their television viewing profile reported either that children in high exposure categories had lower scores on a particular vocabulary test (Plancoulaine et al., 2017; Kim & Chung, 2021; Stockdale et al., 2022) or that there was no significant difference between the different exposure groups (Plancoulaine et al., 2017; Barr et al., 2010; Bittman et al., 2011; Kim & Chung, 2021; Bosma & Blom, 2020; Stockdale et al., 2022). Of the studies that measured overall screen time as a continuous variable, two reported negative associations with generalised vocabulary (Pagani et al., 2013; Carson et al., 2017), while six reported no significant association after controlling for demographic factors (Zhang, Adamo, et al., 2022; Kühhirt & Klein, 2020; Hu et al., 2020; Blankson et al., 2015; Schmidt et al., 2009; Allen et al, 1992). In studies where some or all of the viewing time data was collected before the age of three, one reports a negative relationship between viewing and vocabulary (Pagani et al., 2013), four find no effect (Schmidt et al., 2009; Barr et al., 2010; Bittman et al., 2011; Kühhirt & Klein, 2020), and three find negative effects on some vocabulary measures and no effects on others (Plancoulaine et al., 2017; Kim & Chung, 2021; Stockdale et al., 2022). None of these studies reported positive associations between viewing before the age of three and vocabulary.

Where studies examined subcategories of viewing (e.g. viewing particular genres), results were more inconsistent. For example, a number of studies reported mixed results with positive associations between generalised vocabulary and viewing educational programmes (Wright et al., 2001) and positive effects of co-viewing (Bittman et al., 2011), alongside negative effects for cartoons (Wright et al., 2001) and background television (Bittman et al., 2011). Farangi and Mehrpour (2022a) report that background television exposure is negatively associated with vocabulary for children from high SES backgrounds but that the opposite pattern is observed for children from low SES backgrounds. Rice et al. (1990) report that viewing *Sesame Street* was significantly associated with increased PPVT score but that there was no relationship between vocabulary and viewing non-informative programmes. Finally, Barr et al. (2010) report a negative association between exposure to adult-directed television and vocabulary but no effect of child-directed television.

In summary, where measures of total video screen time were used, these had null or negative associations with vocabulary level. However, results were more variable when looking at specific aspects of video viewing, with some activities (e.g. viewing educational media or co-viewing with a parent) associating with higher vocabulary scores.

##### General language ability

Nine observational studies (29%) opted to use measures which focus on the general language level of a child. This was almost always assessed using standardised language screening tools such as the *Test of Early Language Development* (TELD, or the verbal IQ composite from the *Wechsler Preschool and Primary Scale of Intelligence* (WPPSI-III). Other general language measures included expressive and receptive language questions developed for the Kids in Taiwan longitudinal study (Yu et al., 2022), and measures of the quantity of verbal utterances during a language sample (Farangi & Mehrpour, 2022b).

Of studies that measured total screen time, one reported a positive association between language ability and overall television viewing measured both at age three and at age five (Law et al., 2018). Across five studies, there were seven negative associations reported between overall video exposure and measures of general language abilities including two that tested associations with receptive and expressive language separately (Plancoulaine et al., 2017; McKean et al., 2015; expressive language only: Yu et al., 2022; separate expressive and receptive language measures: Ozyurt & Dinsever, 2017; Hill et al., 2020). Three studies report a null association between overall video exposure and general language measures (Zhang, Weibe, et al., 2022; Arraf, 1990; receptive language only: Yu et al., 2022).

Results for studies that measured subcategories of viewing were again mixed. Farangi and Mehrpour (2022b) divided children according to their family SES and their exposure to background television and found that general language scores were best in the high SES, high background television group, but only for a subset of their measures. Arraf (1990) reported mixed effects of different genres with general language scores increasing with exposure to educational television and decreasing with exposure to commercial television. Finally, Zhang, Weibe, et al. (2022) used a binary variable that captured whether or not children were meeting screen-time recommendations and found better performance in children who did meet the recommendations.

Results for general language measures are therefore more inconsistent than for vocabulary outcomes. While overall screen time is most commonly negatively associated with general language measures, results are more varied for subcategories of viewing and there are some reported positive associations between viewing and language.

##### Syntax or morphology

Six observational studies (19.4%) used measures relating to syntactic or morphological knowledge. In four cases this involved collecting samples of children’s spoken language and analysing them for syntactic complexity (Selnow, 1978; Selnow & Bettinghaus, 1982) or diversity/number of particular syntactic categories such as path verbs (e.g. ‘come’ or ‘go’, Wu et al. 2022) or interrogatives (e.g. questions and question words such as ‘what’, Li et al., 2017). The only other syntactic measure used was a Sentence Repetition Test in which children are asked to repeat sentences of increasing grammatical complexity (i.e. longer sentences that contained more clauses, Oryadi-Zanjani et al., 2021). Bosma & Blom (2020) measured morphological development (the only study to do so) using a test in which children had to produce plural and past participle forms of test items. Of the studies that measured overall video exposure, one reported a positive effect of viewing on the number of interrogative forms used (Li et al., 2017), one reported a negative association between viewing and a sentence repetition test (Oryadi-Zanjani,et al., 2021), and four report null associations with syntactic or morphological measures (syntactic complexity: Selnow, 1978; Selnow & Bettinghaus, 1982; diversity of path verb types: Wu et al., 2022; morphology: Bosma & Blom, 2020). Both Selnow (1978) and Selnow and Bettinhaus (1982) also broke down viewing time into five genre categories and reported non-significant associations between viewing and syntax for all categories (note that this excludes cartoon viewing time in Selnow and Bettinghaus (1982) where a significant association is reported, but this is inconsistently presented as positive and negative in the text). Finally, Wu et al. (2022) report a positive association between parents’ viewing time with children and the quantity of path verb types that children produce. Once again, these findings are inconsistent and do not reveal a clear picture of an association between video exposure and syntax or morphology.

##### Conceptual knowledge

Wu et al. (2022) measured the semantic density of utterances produced by children during the study (i.e. the amount of semantic information contained within the verb phrases) and found no association with total video exposure.

## Discussion

### Main findings

Although there is considerable variability in this field of research, there are a number of patterns that emerge within each study type. Beginning with experimental studies, one key finding is that children do appear to be capable of learning specific words that are embedded in video media. Evidence of this learning comes from comparison of pre- and post-test scores on outcome measures, or a comparison of scores from a video-exposure group with a control group that were not exposed to the target stimuli. Furthermore, comparisons of different media types tended to find no significant difference between the amount learned through viewing compared to the amount learned through shared-reading or listening to radio. Another, perhaps surprising, finding is that there were very few stimuli or contextual factors that appeared to consistently modulate language learning from video. While individual studies reported effects of stimuli and contextual factors, findings across studies were often conflicting (particularly with regard to interactivity and participatory language).

In terms of observational studies, we once again see variability in findings, with mostly null or negative associations between overall video exposure and language but more varied findings when viewing was broken down into more specific categories. In particular, educational content was associated with higher scores on language measures in a number of studies while cartoons and commercial TV were linked to poorer scores.

Another key finding that has emerged across the entire set of studies is the tension between benefits of video for learning specific language structures in the short-term and null or negative effects for global language acquisition in the long-term: the majority of longitudinal association studies report a negative or null relationship between reported TV time and language (Figure 9), while many experimental studies report a significant gain in specific language skills following a viewing session.

While the discrepancy in findings may be partially attributable to methodological differences between experimental and observational approaches, it is also possible that these findings reflect a real paradox in which children appear to benefit from individual exposures to language in video, but long-term exposure may be associated with lower language levels. This phenomenon will be discussed in terms of 1) video content and viewing context, 2) targeted vs generalised outcome measures, and 3) limitations of included studies, including unmeasured confounding.

#### Video content and viewing context: The importance of quality

Inconsistent findings in this field could stem from differences in video content and viewing context. The experimental studies included here tend to use educational or high-quality video stimuli that are almost exclusively designed for children. Often stimuli are designed to include difficult target words (Rice & Woodsmall, 1988) or are chosen because they contain a particular narrative style or structure (Linebarger & Piotrowski, 2009), or participatory cues in which an on-screen character interacts with the viewer (Goldstein et al., 2022). Additionally, the viewing context in experimental studies is often highly controlled with children watching in small groups in a lab or classroom and almost always in the presence of an adult who may help to keep them focused on the video. Figures 6 and 7 shows the number of stimuli and contextual factors that are controlled in these studies. As such, the experimental literature tells us mainly about the impact of viewing *high-quality child-directed* video on language skills. By contrast, observational studies are often less restricted in the type of media that they examine. In some cases, parents simply report the amount of time they think their child spends watching TV or video on any device without any further contextual information (Hill et al., 2020). Although it was not uncommon for researchers to measure additional details about the child’s viewing experience (including information about the content and target audience of the video, whether the TV was just on in the background, and whether anyone else was present), these factors were not controlled and sometimes revealed that children were watching content of more variable quality than the stimuli of experimental studies. As such, observational studies may reveal more about the impact of *naturalistic exposure* that may involve settings or video content that are less conducive to learning.

Taken together, these findings highlight the crucial importance of considering video *quality* and viewing *context* when assessing the impact of video media on children’s linguistic development. The importance of these factors is highlighted by the observational studies that report positive associations between language and viewing educational videos (Wright et al., 2001; Rice et al., 1990; Arraf, 1990) or co-viewing videos with an adult (Bittman et al., 2011). Video quality can also be understood in terms of Fisch’s Capacity Model (Fisch, 2000) which states that children will learn best when educational content is embedded in a comprehensible narrative that can be easily processed by viewers. This is in keeping with the findings of many of the experimental studies showing that children were able to learn new vocabulary when viewing the educational, age-appropriate videos selected or created by researchers. This model is further supported by studies revealing better recall of content that is central to the plot (e.g. Calvert et al., 1982) and better recall for programmes that were child-directed and entertaining (e.g. Lorch et al., 2000). There is some evidence that word learning is also more successful in more engaging videos, with children learning more from participatory (Neuman, Flynn, et al., 2020) and narrative (Linebarger & Piotrowski, 2010) videos rather than expository videos (although note that Neuman, Flynn, et al. also report worse performance on a narrative condition compared to an expository condition).

Although the experimental studies tested the effects of a broad range of stimuli and contextual factors, these were rarely comparisons between high- and low-quality viewing (i.e. both conditions tended to include age-appropriate stimuli and quiet viewing environments). Therefore, while these experimental studies offer useful information about the impact of high-quality video viewed in carefully controlled settings, it is also important to experimentally test the effects of low-quality programmes and viewing scenarios if we are to understand the real-world impact that TV and video are having on children’s development.

It is also important to note that the majority of studies in this review did not examine the *linguistic* quality of the programmes children were watching. It was rare for observational studies to describe the language content of programmes and descriptions in experimental studies were often limited to only those language features that were manipulated across conditions. Linguistic content could potentially be an important feature of programme quality since it likely plays a role both in children’s opportunities to encounter new linguistic structures, and also their ability to comprehend the programme as a whole.

#### Outcome measures: Targeted vs generalised

The different outcome measures used in the two approaches may also drive different findings. As shown in Figure 5, the majority of experimental studies used targeted measures of vocabulary or narrative recall that tested children’s knowledge of language content that they had recently encountered in videos. Where pre- and post-tests were used, this usually resulted in findings of significant improvements following exposure to video (e.g. Rice & Woodsmall, 1988). Although some experimental studies reported effects that remained after a delay (see Samudra, Wong, et al., 2019), tests usually occurred soon after exposure. By contrast, observational studies most commonly used standardised measures that did not deliberately target language content that children had recently encountered. These studies frequently reported null or negative associations between quantity of exposure and language skill, over longer timeframes (e.g. Plancoulaine et al., 2017). In using these generalised measures of language, observational studies are aiming to answer fundamentally different questions to experimental studies (which mainly use targeted measures). Targeted measures allow us to answer important questions about what can be learned in a given viewing scenario, but generalised measures allow for a broader assessment of the impact of video viewing as contextualized within a child’s broader lifestyle.

This difference is particularly important in the context of the displacement hypothesis (discussed in Neuman, 1988; and Roberts et al., 1993) which states that TV time may negatively impact a child’s cognitive skills, not because it is inherently damaging, but because it takes away time from other more beneficial activities. In order to test this theory, we need to understand both the immediate impact of naturalistic television viewing on the learning of specific language structures (as measured by targeted outcomes), but also the longer-term impact of habitual viewing – especially of low-quality content – on language competence (as measured by generalised outcomes). In categorizing the outcome measures as targeted or generalised, we have observed a pattern that is consistent with the predictions of the displacement hypothesis: children are able to learn and gain target language skills during a well-controlled, high-quality viewing session (as seen in the experimental findings reported here), but the video content they view habitually often has a negative or null association with their general language skills (as seen in observational findings). Although the broad findings of this review are consistent with predictions of the displacement hypothesis, a deeper understanding of when and how this effect manifests requires further investigation. For example, under this hypothesis we might expect that impact of video media on language would be different for children who come from different linguistic environments: it might be that television is beneficial for children from impoverished linguistic environments, but harmful for children with access to more enriching language environments (e.g. families with a rich home literacy environment). Without assessing the quality of both the language content within the video media and the ‘baseline’ linguistic environment that might be displaced, we cannot predict when (and for whom) video media might have benefit or cause harm.

#### Limitations of included studies

Finally, the included studies face methodological issues and limitations that may impact the validity and reliability of their findings. Experimental studies typically rely on smaller samples (Mean N = 121 compared to N = 750 for observational studies) most frequently consisting of typically developing children from medium/high SES backgrounds (although some studies deliberately recruited children from low-income backgrounds or children with developmental disorders). This impacts the extent to which we can generalise the results to more diverse groups who may be most at risk of low language. As discussed above, experimental studies also often rely on targeted tests of language knowledge which typically contain relatively small numbers of items and therefore may not be reliable or generalisable. Where studies have looked at item characteristics such as word type, they have found that different parts of speech are learned at different rates (Oetting et al., 1995). This highlights the importance of considering item-level characteristics when developing a bespoke targeted measure of language skills. Another key limitation is ecological validity. As mentioned above, experimental studies typically expose children to high-quality stimuli in an unusual viewing setting (such as a lab or unused room in a school). Ecological validity is particularly important when trying to understand the impact of viewing context on learning. The surprising finding that co-viewing appears to have only limited benefits may reflect the fact that children were either co-viewing with an unfamiliar researcher (Samudra et al., 2020; Neuman, Samudra, et al., 2020) or with a parent who had been instructed to use specific co-viewing techniques (Strouse et al., 2013). Future research should try to assess the impact of more naturalistic co-viewing scenarios that children are likely to experience at home.

For observational studies, key methodological issues include measure validity and confounding. The measures used to assess video exposure typically did not include much (if any) further information about content or viewing context. In order to unpick the complexities of the relationship between viewing and language, researchers should move away from measuring video viewing as a homogeneous activity and instead use measures that are sensitive to the quality of the video and/or viewing environment. The measures of TV exposure used by the studies in this review may also be confounded with other important factors that are thought to impact language skills such as SES, maternal education, and the home literary environment (Ronfani et al., 2015, Schmitt et al., 2011) meaning that the underlying cause of any association with language skills will be hard to determine. Indeed, where these factors have been controlled in statistical models, negative associations tend to reduce or even disappear (Schmidt et al. 2009). Another important factor to consider in any association study looking at child language is genetic confounding (Hart et al., 2021). It could be that parental language ability influences both the home environment (including TV time) and child language outcomes, meaning that associations between TV time and child language may be misleading if parental language skills are not controlled. Of 93 studies, only Schmidt et al. (2009) directly controlled for parental language skills. In this case, the negative association between weekly TV hours in infancy and vocabulary at age three was no longer significant after controlling for maternal education and maternal vocabulary score.

### Similarities to previous reviews

The main finding of inconsistent effects across different study types and designs is in keeping with previous reviews on the topic. For example, Madigan et al. (2020) report that background television exposure is negatively associated with language skills, while high-quality viewing is associated with stronger language skills. Similarly, Kostyrka-Allchorne et al. (2017) find that cognitive outcomes depend on characteristics of the child and features of television exposure. The results reported here are also consistent with a recent meta-analysis looking at the effect of screen media exposure on vocabulary skills only (Jing et al., 2023). In both cases, quality of video media, specificity of outcome measures, and media interactivity were identified as important factors influencing findings about vocabulary. The present review extends this work by additionally identifying factors that have been found to influence other language outcomes including comprehension, syntax, semantics, and general language measures. These findings should inform future meta-analyses focusing on the impact of video media on a broader range of language outcomes.

### Implications and future directions

There are a number of gaps in the literature which should be addressed by future research. Firstly, there is a need for more experimental work which aims to understand the impact of naturalistic video stimuli and viewing conditions on language outcomes. Although many of the included studies used real children’s TV episodes as stimuli, these were often carefully selected, high-quality videos that were designed to be educational (such as *Sesame Street* or *Dora the Explorer*). Future experimental work should try to identify the programmes that are currently popular with children and then carefully test what they are learning about language from these exposures. There is also a need for more detailed observational studies in which more data is collected about the quality of the video content and the context in which it is viewed. For example, there would be great value in expanding the work of Selnow and Bettinghaus (1982) who asked parents to report exactly what their children were watching and then analysed the complexity of the language in those programmes. This data was then used to inform later analyses of the relationship between viewing time and language outcomes. If researchers can develop methods to quantify the quality of children’s viewing experience, this may help to untangle the positive and negative impacts of TV viewing on children’s language, particularly if incorporated within a familial control design where parental language skills are included in statistical models (Hart et al., 2021). More generally, the results of this review have revealed the numerous methodological choices that researchers of this topic must make, and have highlighted choices relating to 1) video stimuli (Are stimuli high quality? Are they naturalistic?), 2) measures of screen time (Are they accurate? Are they sensitive to quality factors?), 3) outcome measures (What is the scope of the measure? Is the measure valid and generalisable?).

### Limitations of this review

The goal of this review was to provide a comprehensive map of the approaches that researchers have used to examine the impact of exposure to language in video on language development. Due to the broad nature of the question, it was necessary to conduct a scoping review rather than a systematic review or meta-analysis. For this reason, studies were not systematically assessed for quality or publication bias. The findings of included studies should therefore be interpreted with caution. Similarly, since effect sizes were not analysed, this review does not provide information about the relative impact of different participant, stimuli, and contextual factors on outcomes. Instead the findings presented here can be used as a basis for future meta-analyses that are interested in the size of these effects.

## Conclusion

The findings of this review reveal huge variation in the approaches and findings of studies linking exposure to language in video to linguistic outcomes. Specifically, there is a divide between highly controlled experimental studies that report primarily positive effects of video media on language, and larger association studies which typically report negative or null effects of video exposure on language. These findings are broadly consistent with Fisch’s Capacity Model (which predicts positive learning outcomes for high quality video) and the displacement hypothesis (which predicts negative impacts only when video displaces more beneficial activities in the long-term). The variability in reported findings can also be understood in terms of the different aims and methodological choices made by experimental and observational researchers: experimental studies are often set up to test children’s ability to learn specific language structures from high-quality child-directed media; observational studies, on the other hand, aim to assess how naturalistic viewing situated in children’s lives associates with their language abilities more generally. Experimental studies are primarily limited by a lack of ecological validity. Observational studies face problems of confounding and imprecise measures of media exposure, which limits our ability to draw any causal conclusions about television viewing and its impact on child language. While television viewing is not a substitute for other enriching activities such as shared book reading, it appears that children can learn new language forms when they are exposed to these through video, and that high-quality, child-directed television may benefit language development across study types.

## Data accessibility statement

Data extracted from included papers can be found at https://osf.io/h43yc/?view_only=438169a9e2ea44648d3fc2a304bc491f.

## Appendix I – Search strategy

The final search was conducted on 27/02/23. Searches for each database used the following search terms (with search parameters for each term set as appropriate for each database):

- “linguistic development” OR “language acquisition” OR “language development” OR “language learning” OR “language outcome*” OR vocabulary OR “word learning” OR syntax OR “sentence comprehension”
- AND “screen media” OR “video media” OR “screen use” OR “media us*” OR “media exposure” OR “TV” OR television
- AND child* OR “toddler*” OR “primary school” OR “primary education” OR “preschool*” OR “kindergarten*” OR “elementary school” OR nurser*

No restrictions were placed on date of publication within any database. Search details for each database are given below, including total number of results, as well as search parameters and results for each search term (where available).

### PsycInfo

Total results: 312

Date range: 1806 to February Week 3 2023

Parameters for each term: [mp=title, abstract, heading word, table of contents, key concepts, original title, tests & measures, mesh word]

**Table:**

LINE	SEARCH TERM	TOTAL RESULTS

1	“language outcome*”	971

2	vocabulary	43108

3	“word learning”	3305

4	syntax	12118

5	“sentence comprehension”	5082

6	“language learning”	18001

7	“language acquisition”	10118

8	“language development”	38562

9	“linguistic development”	737

10	1 or 2 or 3 or 4 or 5 or 6 or 7 or 8 or 9	103103

11	“screen media”	267

12	“video media”	64

13	Television	21302

14	“screen use”	183

15	“media us*”	6165

16	“media exposure”	3040

17	TV	11314

18	11 or 13 or 13 or 14 or 15 or 16 or 17	33228

19	child*	889668

20	“primary school”	13473

21	“primary education”	2196

22	preschool*	115457

23	kindergarten*	22362

24	toddler*	12978

25	nurser*	5472

26	“elementary school*”	75133

27	19 or 20 or 21 or 22 or 23 or 24 or 25	938596

28	10 and 18 and 26	312

### MedLine

Total results: 159

Date range: 1946 to February 27, 2023

Parameters for each item: [mp=title, book title, abstract, original title, name of substance word, subject heading word, floating sub-heading word, keyword heading word, organism supplementary concept word, protocol supplementary concept word, rare disease supplementary concept word, unique identifier, synonyms, population supplementary concept word, anatomy supplementary concept word]

**Table:**

LINE	SEARCH TERM	TOTAL RESULTS

1	“language outcome*”	1078

2	vocabulary	23374

3	“word learning”	1953

4	syntax	6006

5	“sentence comprehension”	1448

6	“language learning”	2365

7	“language acquisition”	3030

8	“language development”	21303

9	“linguistic development”	274

10	1 or 2 or 3 or 4 or 5 or 6 or 7 or 8 or 9	51326

11	“screen media”	238

12	“video media”	39

13	Television	22879

14	“screen use”	386

15	“media us*”	8649

16	“media exposure”	1491

17	TV	16483

18	11 or 12 or 13 or 14 or 15 or 16 or 17	45442

19	child*	2722374

20	“primary school”	11354

21	“primary education”	2616

22	preschool*	993094

23	kindergarten*	8054

24	toddler*	14122

25	“elementary school*”	11912

26	19 or 20 or 21 or 22 or 23 or 24 or 25	2730428

27	10 and 18 and 26	159

### ERIC

Total results: 437

Search syntax:

noft(“linguistic development” OR “language outcome*” OR vocabulary OR “word learning” OR syntax OR “sentence comprehension” OR “language learning” OR “language acquisition” OR “language development”) AND noft(“screen media” OR “video media” OR television OR “screen use” OR “media us*” OR “media exposure” OR TV) AND noft(child* OR “primary school” OR “primary education” OR preschool* OR kindergarten* OR toddler* OR nurser* OR “elementary school*”)

### LLBA

Total results: 224

Search syntax:

noft(“linguistic development” OR “language outcome*” OR vocabulary OR “word learning” OR syntax OR “sentence comprehension” OR “language learning” OR “language acquisition” OR “language development”) AND noft(“screen media” OR “video media” OR television OR “screen use” OR “media us*” OR “media exposure” OR TV) AND noft(child* OR “primary school” OR “primary education” OR preschool* OR kindergarten* OR toddler* OR nurser* OR “elementary school*”)

### Scopus

Total results: 385

Search syntax:

(TITLE-ABS-KEY (“linguistic development” OR “language outcome*” OR vocabulary OR “word learning” OR syntax OR “sentence comprehension” OR “language learning” OR “language acquisition” OR “language development”) AND TITLE-ABS-KEY (“screen media” OR “video media” OR television OR “screen use” OR “media us*” OR “media exposure” OR tv) AND TITLE-ABS-KEY (child* OR “primary school” OR “primary education” OR preschool* OR kindergarten* OR toddler* OR nurser* OR “elementary school*”))

### Web of Science

Total results: 480

Search syntax:

((ALL=(“linguistic development” OR “language outcome*” OR vocabulary OR “word learning” OR syntax OR “sentence comprehension” OR “language learning” OR “language acquisition” OR “language development”)) AND ALL=(“screen media” OR “video media” OR television OR “screen use” OR “media us*” OR “media exposure” OR TV)) AND ALL=(child* OR “primary school” OR “primary education” OR preschool* OR kindergarten* OR toddler* OR nurser* OR “elementary school*”)

**Total across all sources = 1997 records**
